# New species of Trigonalyidae (Hymenoptera) from NW China

**DOI:** 10.3897/zookeys.698.13366

**Published:** 2017-09-18

**Authors:** Jiang-Li Tan, Cornelis van Achterberg, Qing-Qing Tan, Lin-Peng Zhao

**Affiliations:** 1 Shaanxi Key Laboratory for Animal Conservation / Key Laboratory of Resource Biology and Biotechnology in Western China, College of Life Sciences, Northwest University, 229 North Taibai Road, Xi’an, Shaanxi 710069, China; 2 Shaanxi Changqing National Nature Reserve, Changqing Jiayuan, 176 Dongyi Huan Road, Hanzhong, Shaanxi 723000, China

**Keywords:** Trigonalyidae, *Bareogonalos*, *Orthogonalys*, *Jezonogonalos*, *Taeniogonalos*, *Teranishia*, new species, new record, biology, *Vespula structor*, China, Shaanxi, Ningxia

## Abstract

Four new species of Trigonalyidae are described and illustrated from Qinling Mts (Shaanxi, NW China): *Bareogonalos xibeidai* Tan & van Achterberg, **sp. n.**, *Jezonogonalos mandibularis* **sp. n.**, *J. shaanxiensis* **sp. n.**, and *Taeniogonalos paraclypeata* **sp. n.** *Orthogonalys hirasana* Teranishi, 1929, is re-instated and reported from China. The female of *Taeniogonalos alticola* (Tsuneki, 1991) is described for the first time. In total, 18 species are known from Shaanxi province, 20 species for NW China, and eight described species are newly recorded for Shaanxi: *Jezonogonalos luteata* Chen et al., 2014, *Orthogonalys hirasana* Teranishi, 1929, *O. elongata* Teranishi, 1929, *Pseudogonalos hahnii* (Spinola, 1840), *Taeniogonalos alticola* (Tsuneki, 1991), *T. formosana* (Bischoff, 1913), *T. taihorina* (Bischoff, 1914), and *Teranishia glabrata* Chen et al., 2014. *Poecilogonalos maga* Teranishi, 1929, **syn. n.** and *Taiwanogonalos claripennis* Tsuneki, 1991, **syn. n.** are new synonyms of *Taeniogonalos taihorina* (Bischoff, 1914) and *Taiwanogonalos alishana* Tsuneki, 1991, **syn. n.** of *Taeniogonalos alticola* (Tsuneki, 1991). Revised keys to species of the genera *Bareogonalos*, *Jezonogonalos*, and *Orthogonalys* are included.

## Introduction

Trigonalyidae (Hymenoptera) is a worldwide small family in its own superfamily Trigonalyiodea, with 115 recognized species (Carmean and Kimsey 1998; Smith and Stocks 2005; Santos et al. 2012; Smith and Tripotin 2012; Smith et al. 2012, 2015; Chen et al. 2014; Yamane 2014). Most species of this family occur in tropical and subtropical regions and the family is absent in arctic and alpine habitats (Carmean and Kimsey 1998). Surprisingly, Trigonalyidae are fairly common at 1300-1500 m altitude in the Qinling Mts of Shaanxi (NW China). In total, 18 species from Shaanxi are recorded in this paper, which is 41% of the 44 spp. known from China.

Trigonalyidae are often misidentified; slender specimens (especially of *Orthogonalys*) with white banded antennae are mistaken for Ichneumonidae and robust specimens with black antennae for aculeate wasps (e.g., of the family Crabronidae). They can be identified by the combination of the well-developed costal cell of the fore wing and the presence of unequal mandibles with 3–5 large teeth (Figs 10, 36, 57) and plantar lobes on the tarsal segments (Figs 12, 29, 91). In addition, the tarsal claws are cleft (bifurcate with the inner tooth larger than the outer one: Figs 24, 86) and as pointed out in Carmean and Kimsey (1998) the females have sparse white scales or specialized seta on the outside of the middle antennal segments.

Most Trigonalyidae develop as a hyperparasitoids on parasitoid wasp or fly larvae inside caterpillars and sawfly larvae. Primary endoparasitism of sawflies occurs, but the parasitoid still acts facultatively as a hyperparasitoid (Yamane and Terayama 1983; He and Chen 1986; Weinstein and Austin 1991; Carmean and Kimsey 1998). Up to more than 2000 eggs may be laid on leaves, which are eaten eventually by caterpillars and sawfly larvae. In the digestive tract the eggs hatch, the mobile larva bores through the intestine wall to search for an eventually present parasitoid wasp (Ichneumonidae or Braconidae) or fly (Tachinidae). Other species are brought into the nests of social Vespidae because they are inside the caterpillars used as prey by the wasps. Inside the nest they develop as primary endoparasitoids of the social wasp larvae.

This paper is an addition to the revision of the Chinese fauna of the family Trigonalyidae by Chen et al. (2014). In that revision 40 species were treated belonging to eight genera: *Bakeronymus* Rohwer, 1922; *Bareogonalos* Schulz, 1907; *Jezonogonalos* Tsuneki, 1991; *Lycogaster* Shuckard, 1841; *Orthogonalys* Schulz, 1905; *Pseudogonalos* Schulz, 1906, *Taeniogonalos* Schulz, 1906, and *Teranishia* Tsuneki, 1991. Up to now, five of the genera and 13 spp. are recorded from NW China, of which two genera and six species are known from Shaanxi. In this paper eight described species are added as new for Shaanxi (*Jezonogonalos luteata* Chen, van Achterberg, He & Xu, 2014, *Orthogonalys hirasana* Teranishi, 1929, *O. elongata* Teranishi, 1929, *Pseudogonalos hahnii* (Spinola, 1840), *Taeniogonalos alticola* (Tsuneki, 1991), *T. formosana* (Bischoff, 1913), *T. taihorina* (Bischoff, 1914), and *Teranishia glabrata* Chen, van Achterberg, He & Xu, 2014), and an additional four species from Shaanxi are new to science. The result is six genera (+20%) and 20 species (+54%) for NW China, and six genera (+200%) and 18 species (+200%) for Shaanxi (Table 1).

**Table 1.** List of Chinese species of the family Trigonalyidae Cresson in NW China after this study. An asterisk indicates a new record.

Species	Distribution in China
*Bareogonalos xibeidai* sp. n.	*Shaanxi (Palaearctic)
*Jezonogonalos luteata* Chen et al., 2014	*Shaanxi, (Palaearctic), Sichuan (Oriental)
*Jezonogonalos mandibularis* sp. n.	*Shaanxi (Palaearctic)
*Jezonogonalos shaanxiensis* sp. n.	*Shaanxi (Palaearctic)
*Orthogonalys clypeata* Chen et al., 2014	Shaanxi, Ningxia (Palaearctic), Guizhou, Sichuan, Yunnan (Oriental)
*Orthogonalys elongata* Teranishi, 1929	*Shaanxi, Henan (Palaearctic), Sichuan, Tibet (Oriental)
*Orthogonalys hirasana* Teranishi, 1929, re-instated	*Shaanxi (Palaearctic), *Sichuan (Oriental)
*Orthogonalys paraclypeata* sp. n.	*Shaanxi (Palaearctic), Sichuan, Tibet (Oriental)
*Orthogonalys robusta* Chen et al., 2014	Shaanxi (Palaearctic), Guangxi (Oriental)
*Pseudogonalos hahnii* (Spinola, 1840)	*Shaanxi, Inner Mongolia, Liaoning, Beijing, Hebei, Henan (Palaearctic), Yunnan (Oriental)
*Taeniogonalos alticola* (Tsuneki, 1991)	*Shaanxi, *Ningxia (Palaearctic)
*Taeniogonalos bucarinata* Chen et al., 2014	Shaanxi, Ningxia, Gansu, Henan (Palaearctic), Zhejiang, Fujian, Sichuan, Yunnan (Oriental)
*Taeniogonalos fasciata* (Strand, 1913)	Shaanxi, Jilin, Liaoning, Henan, Anhui (Palaearctic), Zhejiang, Taiwan, Fujian, Hunan, Guangdong, Guangxi, Hainan, Guizhou (Oriental)
*Taeniogonalos formosana* (Bischoff, 1913)	*Shaanxi, Ningxia, Shanxi, Henan, Jilin (Palaearctic), Zhejiang, Fujian, Taiwan, Guangdong, Sichuan, Yunnan, Guizhou, Tibet (Oriental)
*Taeniogonalos mongolica* (Popov, 1945)	Inner Mongolia (Palaearctic)
*Taeniogonalos subtruncata* Chen et al., 2014	Shaanxi (Palaearctic)
*Taeniogonalos taihorina* (Bischoff, 1914)	*Shaanxi, Ningxia, Heilongjiang, Gansu (Palaearctic), Zhejiang, Fujian, Taiwan, Hubei, Guangxi, Sichuan, Yunnan, Tibet (Oriental)
*Taeniogonalos tricolor* (Chen, 1949)	Shaanxi, Henan (Palaearctic), Zhejiang, Hubei, Fujian, Jiangxi, Hainan, Guangxi, Sichuan, Guizhou, Yunnan (Oriental)
*Teranishia crenulata* Chen et al., 2014	Ningxia, Gansu (Palaearctic), Sichuan (Oriental)
*Teranishia glabrata* Chen et al., 2014	*Shaanxi, Ningxia, Henan (Palaearctic), Zhejiang, Sichuan (Oriental)

## Materials and methods

The specimens were mainly collected in Malaise traps, but a few by sweep net. The material was stored in 70% ethanol, prepared with the AXA method (van Achterberg 2009; van Achterberg et al. 2010) and glued on card points. Observations and descriptions were made with an Olympus SZX11 stereomicroscope and fluorescent lamps. Photographic images were made with the Keyence VHX-5000 digital microscope and processed with Adobe Photoshop CS5, mostly to adjust the size and background.

Morphology. For other terminology used in this paper, see van Achterberg (1988, 1993), Hong et al. (2011) and Chen et al. (2014). Measurements were taken as indicated by van Achterberg (1988). Additional non-exclusive characters in the key are between brackets.

Material. Types and other examined specimens are deposited in the Northwest University, Xi’an, NW China (**NWUX**) and Naturalis Biodiversity Center, Leiden, Netherlands (**RMNH**).

## Systematics

### Trigonalyidae

Taxon classificationAnimaliaHymenopteraTrigonalyidae

Cresson, 1887

Trigonalyidae Cresson, 1887: 183; Carmean and Kimsey 1998: 54 (as Trigonalidae, corrected by Krieger (1894) to Trigonalyidae); Chen et al. 2014: 9–203 (synonyms, references, diagnosis, keys to genera and spp., revision of Chinese spp.).

#### Notes.

The first part of the name Trigonalyidae refers most likely to protuberance of the second sternite present in females of several spp. (but absent in the species treated in this paper, except *Bareogonalos* Schulz), which is triangular in lateral view.

### Bareogonalos

Taxon classificationAnimaliaHymenopteraTrigonalyidae

Schulz, 1907

Figs 1–2 , 3–13 , 14–22 , 23–28

Bareogonalos Schulz, 1907: 18; Marshakov 1981: 104; Tsuneki 1991: 9; Weinstein and Austin 1991: 412; Carmean and Kimsey 1998: 60. Type species (designated by Schulz 1907): Trigonalys canadensis Harrington, 1896.
Nippogonalos Uchida, 1929: 79; Tsuneki 1991: 4; Lelej 1995: 12; Weinstein and Austin 1991: 412. Type species (by monotypy): Nippogonalos jezoensis Uchida, 1929. Synonymised by Bischoff 1938.
Makotogonalos Yamane, 2014: 18 (proposed as subgenus). Type species (by original designation): Bareogonalos huisuni Yamane & Yamane, 1975.

#### Biology.

Reared from nests of *Vespa*, *Vespula*, *Dolichovespula* and *Provespa* spp. (Vespinae: Vespidae); the larva of at least one species has a final ectoparasitoid phase (Carmean and Kimsey 1998) after the initial endoparasitoid phase. Collected in August–October.

#### Distribution.

East Palaearctic, Nearctic (but intruding Central America by reaching SW Mexico), Oriental.

#### Key to Old World species of the genus *Bareogonalos* Schulz

**Table:** 

1	Metanotum with large protuberance and long setae; left mandible with 2 subapical teeth (Fig. 10); maxillary palp with 6 segments; anterior half of scutellum flat or weakly convex and at same level as mesoscutum or slightly above it (Figs 5, 16, 24); fourth and fifth tergites largely smooth and shiny (Fig. 1); subgenus Bareogonalos Schulz, 1907	**2**
–	Metanotum weakly convex, without protuberance and its setae medium-sized; left mandible with 3 subapical teeth; maxillary palp with 5 segments; anterior half of scutellum distinctly convex and above level of mesoscutum; fourth and fifth tergites largely finely and rather densely punctate and rather dull; [head nearly as wide as mesoscutum]; subgenus Makotogonalos Yamane, 2014	**3**
2	Surrounding membrane of veins 1-SR and 1-M of fore wing yellowish or infuscated as most of wing membrane (Figs 23, 24); width of head in dorsal view 0.8–0.9 times maximum width of mesoscutum; third sternite of ♀ distinctly protruding medio-posteriorly in lateral view (Fig. 26) and apical part usually distinctly exposed in ventral view (Fig. 27); antenna with 21–23 segments (Fig. 28), rarely with 20 segments; mesoscutum with median groove posteriorly (Fig. 25); [very colour variable species (Tsuneki 1991)]; Japan (Hokkaido, Honshu, Kyushu; reared from several *Vespa simillima* nests (Yamane 2014) and of a *Vespula flaviceps karenkona* nest); Russia (Far East) [the specimens reported from Java will be dealt with in a following paper]	***B. jezoensis* (Uchida, 1929)**
–	Surrounding membrane of veins 1-SR and 1-M of fore wing subhyaline as remainder of wing except its apical third (Figs 3, 14); width of head in dorsal view 1.1 times maximum width of mesoscutum (Figs 4, 15); third sternite of ♀ less protruding medio-posteriorly in lateral view (Fig. 8) and obscured by second sternite in ventral view (Fig. 7); antenna with 20–21 segments (Figs 13, 18); mesoscutum without median groove posteriorly (Figs 4, 15); China (Shaanxi; reared from *Vespula structor* nest)	***B. xibeidai* sp. n.**
3	Mesoscutum anteriorly and scutellum partly brownish yellow; basal half of first metasomal tergite black; combined fourth and fifth maxillary palp segments 1.7 times as long as third segment; China (Taiwan)	***B. huisuni* Yamane & Yamane, 1975**
–	Mesoscutum anteriorly and scutellum entirely black; basal half of first metasomal tergite yellowish brown; combined fourth and fifth maxillary palp segments 1.2 times as long as third segment; Indonesia (Sumatra)	***B. provespae* Yamane, 2014**

### Bareogonalos xibeidai

Taxon classificationAnimaliaHymenopteraTrigonalyidae

Tan & van Achterberg sp. n.

http://zoobank.org/6E4EB557-42E1-4269-8263-931C11C1460B

Figs 1–2 , 3–13 , 14–22

#### Type material.

Holotype, ♀ (NWUX) “NW China: Shaanxi, Fengxian County, Baoji, Jialingjiang Riv[er] Source, 1513 m, 34°25'N 106°94'E, 1.ix.2016, JL. Tan & QQ. Tan, NWUX”, “Reared from nest of *Vespula structor* (Smith)”. Paratypes: 6 ♀ + 8 ♂ (NWUX, RMNH), same label data.

#### Diagnosis.

Left mandible with two subapical teeth, ventral tooth shorter than dorsal tooth; maxillary palp with six segments; width of head in dorsal view 1.05–1.10 times maximum width of mesoscutum (Figs 4, 15); anterior half of scutellum flat and at same level as mesoscutum; metanotum with large protuberance and long setae; wing membrane near veins 1-SR and 1-M of fore wing subhyaline as remainder of wing except darkened apical third of wing (Figs 3, 14); hind basitarsus slender (Fig. 12); fourth and fifth tergites largely smooth and shiny; third sternite of ♀ in ventral view distinctly protruding medio-posteriorly (but obscured by second sternite). Differs from the only other known Palaearctic species, *B. jezoensis* (Uchida) mainly by the colour of the wing membrane (largely subhyaline *vs* largely yellowish in *B. jezoensis*; Fig. 23), the median groove of the mesoscutum (absent posteriorly *vs* present) and the wider head (1.05–1.10 times *vs* 0.80–0.90 times width of mesoscutum).

#### Description.

Holotype, ♀, length of body 9.2 mm (of fore wing 8.5 mm).

*Head*. Antenna with 20 segments, segments of apical half 1.1–1.3 times as long as wide (Fig. 13); frons and vertex smooth and strongly shiny, flat except a shallow depression behind each posterior ocellus (Figs 9, 15), with fine and long greyish setae; head gradually narrowed behind eyes and 1.05 times as wide as mesoscutum (Fig. 4); dorsal length of eye 0.9 times length of temple (Fig. 9); temple smooth and shiny; occipital carina thick and moderately lamelliform medio-dorsally and with a short crenula; supra-antennal elevations low, as a thin rim and smooth; clypeus moderately depressed medio-ventrally and flat above depression.

*Mesosoma*. Length of mesosoma 1.1 times its height (Fig. 5); mesopleuron below transverse mesopleural groove densely moderately reticulate-rugose, narrowly smooth posteriorly and with satin sheen, above groove coarsely reticulate; transverse mesopleural groove wide, deep and coarsely crenulate (Fig. 5); notauli medium-sized and distinctly crenulate; mesoscutum coarsely rugose-reticulate and with satin sheen contrasting with shiny head, antero-medially with parallel-sided flattened area, convex but medially slightly depressed (Fig. 4); scutellar sulcus curved, parallel-sided and coarsely crenulate; scutellum reticulate-rugose, convex and anteriorly above level of mesoscutum; metanotum medially with large and coarsely reticulate trapezoid protuberance (Figs 4, 5), apically shallowly emarginate, in lateral view not or hardly protruding over base of propodeum; propodeum largely finely reticulate-rugose, but anteriorly coarsely crenulate (Fig. 4).

*Metasoma*. First tergite 0.3 times as long as apically wide (of paratype, obscured by propodeum in holotype), gradually narrowed basally, flat medially and straight apically; second tergite smooth and strongly shiny as following tergites, but with lateral patch of fine punctures; second sternite rather densely finely punctate and shiny, its medio-apical protuberance densely setose and widely truncate medially (Fig. 7), protuberance of third sternite similar but smaller and obscured by second sternite in ventral view (Fig. 7); down curved apex of metasoma nearly up to protuberance of second sternite (Fig. 8).

*Legs.* Hind coxa and femur smooth and shiny.

*Colour.* Black; palpi, mandible apically, antenna (but scapus yellow ventrally and basal third of antenna brown), tegulae (but humeral plate brown), first tergite, veins (except yellowish veins 1-SR, 1-M and 1-SR+M of fore wing) and pterostigma dark brown; coxae basally black; femora (except yellow anterior face of fore femur, base and apex of middle and hind femora), and inner side of hind tibia (and partly ventrally) dark brown; 3 apical segments of hind tarsus and infuscate; pronotum dorso-apically, pair of wide patches on mesoscutum anteriorly, axilla, scutellum latero-posteriorly, dorso-apical patch of mesopleuron, pair of large antero-lateral patches on second–sixth tergites (touching each other on fourth and fifth tergites), pair of small subposterior patches on first sternite, large V-shaped patch on second sternite, and remainder of legs yellow; membrane of fore wing subhyaline, but apical 0.4 weakly infuscate (Fig. 3).

*Variations.* Length of body 8.0–9.3 mm, of fore wing 8.0–8.9 mm; antenna of ♀ with 20(2) or 21(2) segments; width of head in dorsal view 1.05–1.10 times maximum width of mesoscutum; mesoscutum sometimes entirely black, or with additional yellow spot medio-posteriorly; length of vein 1-M of fore wing 2.1–3.1 times as long as vein 1-SR; mandible entirely blackish brown or with some brownish patches subapically; antero-lateral yellow patches of metasoma either all separated from each other, or all touching medially, but sometimes only on second–fourth tergites being separated; yellow patch of second sternite may be separated into large patches; inner side of hind tibia largely dark brown or only apical third.

*Male.* Length of body 7.2–9.6 mm, of fore wing 6.8–8.0 mm; antenna with 20(4) or 21(1) segments, basal quarter of antenna black, but scapus pale yellow with upper part dark brown and pedicellus brown; mesoscutum, mesopleuron, and metasoma entirely black or mesoscutum with pair of tiny yellow spots anteriorly and second sternite with pair of medium sized yellow spots; veins 1-SR, 1-M and 1-SR+M of fore wing dark brown. Genitalia, see Fig. 22.

#### Biology.

The type series was reared from a nest of *Vespula structor* (Smith, 1870) (Vespidae: Vespinae).

#### Etymology.

Named after one of the oldest universities in China, the Northwest University in Xi’an, for providing us the facilities to research the biodiversity of Qinling Mts. It also points to the fact that this is the most north-western locality the genus is known from (“xibei” means “northwest” in Chinese).

**Figures 1, 2.**
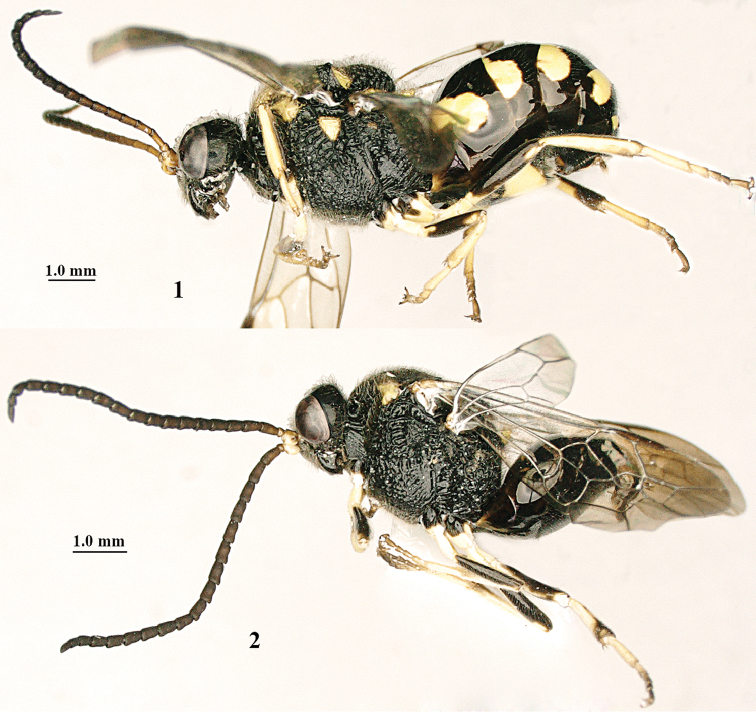
*Bareogonalos xibeidai* sp. n. **1** female holotype, habitus lateral **2** male paratype, habitus lateral.

**Figures 3–13.**
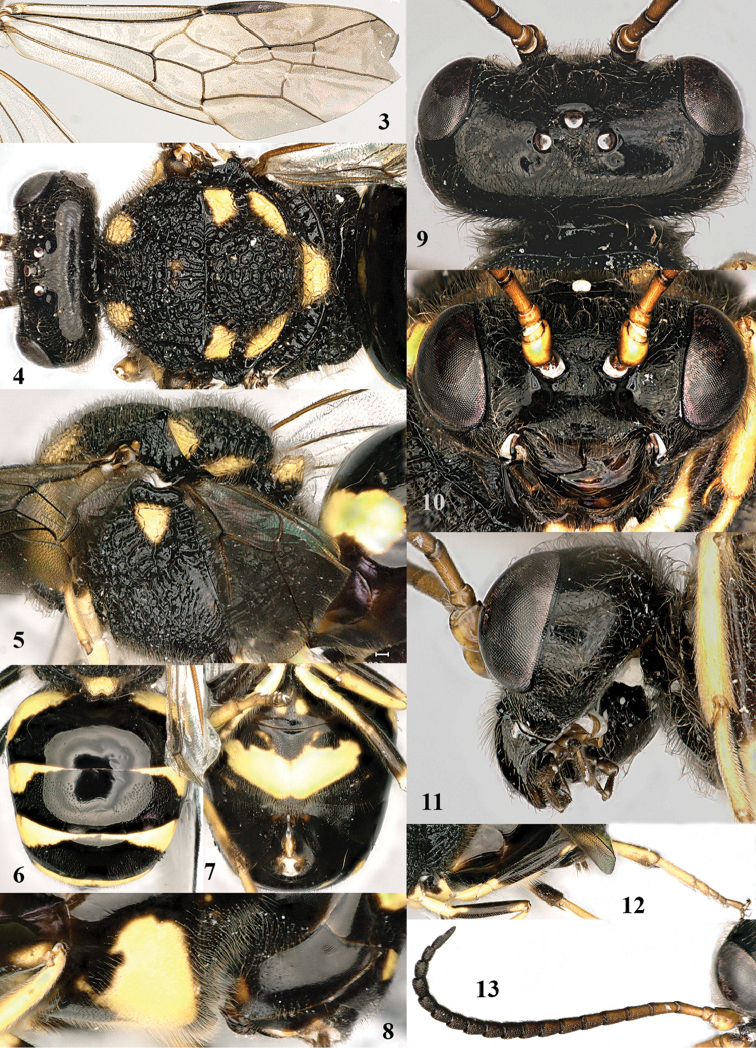
*Bareogonalos xibeidai* sp. n., female, holotype. **3** fore wing **4** head and mesosoma dorsal **5** mesosoma lateral **6** metasoma dorsal **7** metasoma ventral **8** detail of second and third sternites lateral **9** head dorsal **10** head anterior **11** head lateral **12** hind leg lateral **13** antenna lateral.

**Figures 14–22.**
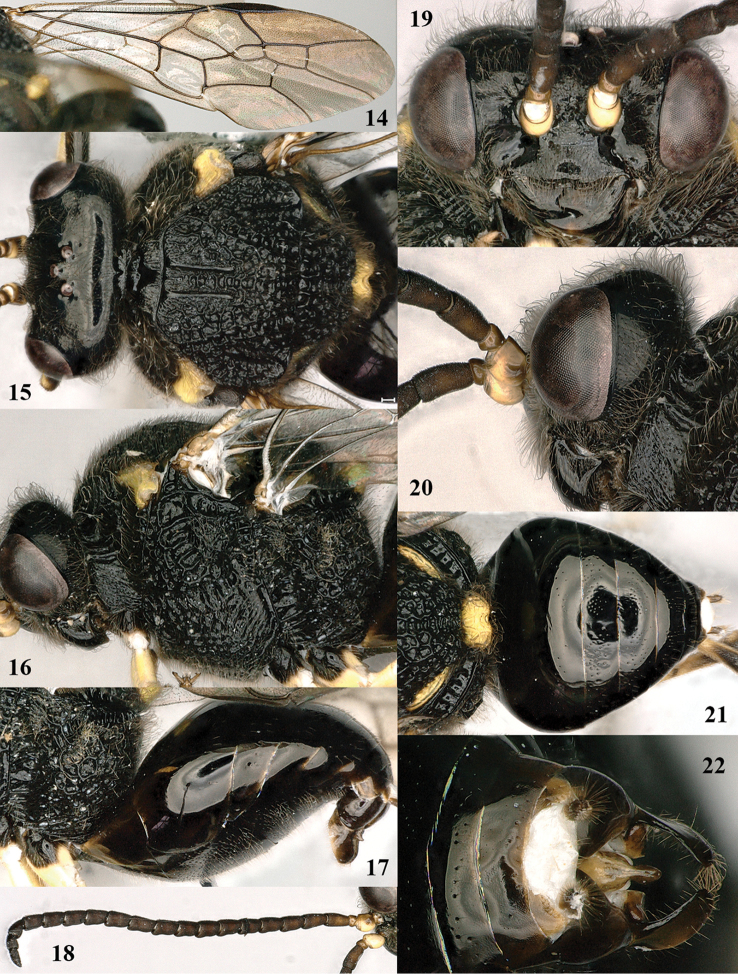
*Bareogonalos xibeidai* Tan & van Achterberg, sp. n., male, paratype. **14** fore wing **15** head and mesosoma dorsal **16** mesosoma lateral **17** metasoma lateral **18** antenna lateral **19** head anterior **20** head lateral **21** metasoma dorsal **22** genitalia dorsal.

### Jezonogonalos

Taxon classificationAnimaliaHymenopteraTrigonalyidae

Tsuneki, 1991

Figs 29 , 30–38 , 39 , 40–48

Jezonogonalos Tsuneki, 1991: 32, 2003: 4; Carmean and Kimsey 1998: 70; Chen et al. 2014: 22–44 (diagnosis, key). Type species (by monotypy): Jezonogonalos marujamanae Tsuneki, 1991 [= J. marujamae Tsuneki, 1991]. Synonymized with Pseudogonalos Schulz, 1906, by Lelej (1995) and re-instated by Chen et al. (2014).

#### Biology.

Unknown. Collected in June–November.

#### Distribution.

China, Japan.

#### Key to species of the genus *Jezonogonalos* Tsuneki

**Table:** 

1	First metasomal tergite about as long as its apical width; supra-antennal elevations only apically ivory; third sternite 0.6–0.7 times as long as second sternite; [metasoma of ♂ entirely black and slender; occipital carina extensively crenulated dorsally and widened; third submarginal cell of fore wing about 0.4 times as long as second submarginal cell]	***J. jiangliae* Chen, van Achterberg, He & Xu, 2014**
–	First tergite 0.6–0.8 times as long as its apical width (Figs 33, 43); at least apical half of supra-antennal elevations yellow or ivory (Figs 35, 45) or entirely black; third sternite 0.1–0.6 times as long as second sternite (Figs 34, 44)	**2**
2	Fore wing subhyaline; metasoma of ♂ with narrow yellowish-brown apical band on all tergites; third sternite about 0.1 times as long as second sternite; [occipital carina only medio-dorsally with short carina]	***J. elliptifera* Chen, van Achterberg, He & Xu, 2014**
–	Fore wing with more or less conspicuous dark brown patch below pterostigma (Figs 30, 40); metasoma of ♂ entirely black dorsally or nearly so; third sternite 0.3–0.6 times as long as second sternite (Figs 34, 44)	**3**
3	Occipital carina extensively crenulated dorsally and present posteriorly; third sternite 0.30–0.40 times as long as second sternite	**4**
–	Occipital carina only anteriorly with short carina medio-dorsally, smooth posteriorly (Figs 35, 45); third sternite 0.35–0.60 times as long as second sternite (Figs 34, 44)	**5**
4	Supra-antennal elevations largely yellow; frons and vertex sparsely and finely punctate; occipital carina moderately wide dorsally	***J. luteata* Chen, van Achterberg, He & Xu, 2014**
–	Supra-antennal elevations black; frons and vertex densely and coarsely punctate; occipital carina very wide dorsally	***J. nigrata* Chen, van Achterberg, He & Xu, 2014**
5	Mesoscutum shiny and largely smooth; propodeum antero-laterally shiny and smooth; frons shiny and with wide smooth interspaces between punctures; mandible yellow except for orange brown teeth; third submarginal cell of fore wing narrower than second submarginal cell	***J. laeviceps* (Tsuneki, 1991)**
–	Mesoscutum rather dull and largely sculptured (Figs 31, 41); propodeum antero-laterally with matt and coriaceous area (Figs 31, 41); frons with satin sheen and with narrow to medium-sized interspaces between punctures; colour of mandible variable, **if** yellow then third submarginal cell of fore wing anteriorly wider than second submarginal cell (Fig. 30)	**6**
6	Third submarginal cell of fore wing anteriorly much narrower than second submarginal cell; mandible and malar space largely dark brown; inner orbita of eye with narrow ivory stripe not reaching malar space; propodeum regularly transversely striate; [supra-antennal elevations black; second sternite with lateral pale patch besides apical margin]	***J. marujamae* Tsuneki, 1991**
–	Third submarginal cell of fore wing anteriorly much wider than second submarginal cell (Figs 30, 40); colour of mandible variable, **if** largely dark brown then malar space largely ivory (Fig. 46); inner orbita of eye variable, **if** with an ivory stripe then stripe continuous with ivory malar space; propodeum with irregular and more or less curved striae or only with coarse rugae	**7**
7	Mandible largely dark brown (Figs 46, 47); scutellum slightly higher than level of metanotum in lateral view (Fig. 39) and medially flattened (Fig. 41); inner orbita of eye conspicuously ivory and continuous with largely ivory malar space (Fig. 46); middle lobe of mesoscutum with pair of lateral elongate ivory patches anteriorly (Figs 41, 42)	***J. shaanxiensis* sp. n.**
–	Mandible pale brown and yellowish or ivory (Figs 36, 37); scutellum protruding far above level of metanotum in lateral view (Fig. 29) and medially slightly longitudinally depressed (Fig. 31); inner orbita of eye largely dark brown, **if** partly ivory then not connected to malar space (Fig. 36); middle lobe of mesoscutum entirely black anteriorly (Figs 31, 32)	**8**
8	Frons spaced punctulate; scutellum largely smooth and shiny; supra-antennal elevations black apically; hind trochanter brownish yellow; mesopleuron medially below precoxal sulcus largely smooth and shiny	***J. satoi* (Tsuneki, 1991)**
–	Frons coarsely and densely punctate (Figs 35, 36); scutellum densely rugose and rather matt (Fig. 31); supra-antennal elevations ivory apically (Fig. 35); hind trochanter ivory and partly infuscated (Fig. 38); mesopleuron medially below precoxal sulcus densely sculptured and rather matt (Fig. 32)	***J. mandibularis* sp. n.**

### Jezonogonalos luteata

Taxon classificationAnimaliaHymenopteraTrigonalyidae

Chen, van Achterberg, He & Xu, 2014

Jezonogonalos luteata Chen et al., 2014: 35–38 (diagnosis, description, distribution).

#### Material.

2 ♂ (NWUX, RMNH): “NW China: Shaanxi, Ningqiang, Hanzhong, Tiankeng, Chanjiyan, N32.46° E106.30°, 25.vi-22.vii.2017, b[lack] Mal. trap, alt. 1638 m, Tan Jiangli, NWUX”.

#### Distribution.

China (*Shaanxi, Sichuan). New for Shaanxi and second record of species.

### Jezonogonalos mandibularis

Taxon classificationAnimaliaHymenopteraTrigonalyidae

Tan & van Achterberg sp. n.

http://zoobank.org/20731817-7795-4678-BE87-A8C625213A29

Figs 29 , 30–38

#### Type material.

Holotype, ♀ (NWUX) “NW China: Shaanxi, Upper Changqing Re[ser]v[e], Shanshuping, 1556 m, 33.67N 107.58E, 25.viii.–22.ix.2016, Y[ellow Malaise] T[rap], Zhao Lin-Peng, NWUX”.

#### Diagnosis.

Occipital carina very wide medio-dorsally, with pair of curved lamellae (Fig. 35); outer side of supra-antennal elevations subvertical and elevations about 0.6 times as long as scapus (Fig. 35); frons largely coarsely punctate (Fig. 36); supra-antennal elevations largely ivory (Fig. 35); mandible mainly pale brown, except its ivory base and dark teeth (Fig. 36); metasoma dorsally largely smooth, largely black with narrow pale apical bands (Figs 29, 33); first tergite about 0.7 times as long as its apical width (Fig. 33); third sternite about 0.3 times as long as second sternite (Fig. 34). Close to *J. satoi* (Tsuneki) from Taiwan, from which is differs mainly because of the coarsely and densely punctate frons (sparsely punctulate in *J. satoi*), the densely rugose and rather matt scutellum (largely smooth and shiny), clypeus with fine acute tooth medio-ventrally (blunt) and the largely ivory supra-antennal elevations (black).

#### Description.

Holotype, ♀, length of body 8.5 mm (of fore wing 7.4 mm).

*Head*. Antenna with 25 segments; frons coarsely punctate (except anteriorly), interspaces narrow and smooth (Figs 35, 36), with rather long setae; vertex largely smooth behind posterior ocelli and posteriorly, but medially punctate and with some short rugae and antero-laterally with oblique rugulae (Fig. 35); temple largely smooth, punctulate, but near mandible punctate (Fig. 37); head gradually narrowed behind eyes, eye in dorsal view 1.4 times as long as temple (Fig. 35); occipital carina strongly widened and pair of circular lamellae medio-dorsally, without distinct carinae (Figs 31, 35); supra-antennal elevations strongly enlarged (about 0.6 times as long as scapus), outer side subvertical and with distinct rugae; clypeus concave and thick medio-ventrally and area above it convex and acutely protruding (Fig. 36).

*Mesosoma*. Length of mesosoma 1.5 times its height (Fig. 32); mesopleuron antero-dorsally densely reticulate-rugose, dorso-posteriorly with some rugosity and remainder smooth and shiny, and antero-ventrally curved rugulose (Fig. 32); notauli wide, deep and distinctly crenulated; middle lobe of mesoscutum transversely rugose and with finer interconnected sculpture, lateral lobes mainly rugose except for a smooth shallow groove (Fig. 31); scutellar sulcus very wide and coarsely crenulated; scutellum densely reticulate-rugose, convex laterally and shallowly depressed medially, in lateral view far above level of metanotum (Fig. 29); metanotum medially evenly convex and finely rugose (Fig. 31); propodeum antero-laterally irregularly rugose and interspaces more or less coriaceous, matt, remainder coarsely transversely rugose and shiny but smooth posteriorly (Fig. 31); posterior propodeal carina thick lamelliform (foramen about twice as wide as high medially).

*Wings.* Fore wing: length of vein 1-M 1.7 times as long as vein 1-SR; third submarginal cell much wider anteriorly than second cell (Fig. 30).

*Metasoma*. First tergite 0.7 times as long as its apical width, smooth but basal depression anteriorly striate (Fig. 33); second and following tergites smooth and shiny; sternites rather densely finely punctate, with wide smooth interspaces; second sternite weakly curved in lateral view; third sternite about 0.3 times as long as second sternite (Fig. 34); hypopygium triangularly protruding in ventral view (Fig. 34).

*Colour.* Black; inner orbita vaguely partly orange brown; malar space and supra-antennal elevations largely ivory; minute patch of outer orbita, vertex posteriorly, occipital carina medially dark brown; apex of scapus and pedicellus, and mandible largely pale brown but basally ivory and teeth dark brown (Figs 36, 37); tegulae and pronotal lobe below it pale yellowish; first metasomal tergite apically broadly ivory and narrower laterally, first and second sternites with large ivory patch apico-laterally; with narrow ivory apical band at apex of sternites and of second and following tergites (Figs 29, 33); palpi rather dark brown; remainder of antenna blackish; fore leg (except black coxa and trochanter) yellowish brown; middle and hind legs mainly dark brown but coxae black (except ivory apex of hind coxa) and hind trochanter ivory with some faint infuscation; pterostigma brownish yellow, but anteriorly (except basally) and apically blackish; basal half of marginal cell and third submarginal cell dark brown and remainder of wing membrane subhyaline (Fig. 30).

*Male.* Unknown.

#### Biology.

Unknown.

#### Distribution.

China (Shaanxi). Collected at 1556 m.

#### Etymology.

Named after its conspicuously coloured mandible.

**Figures 23–28.**
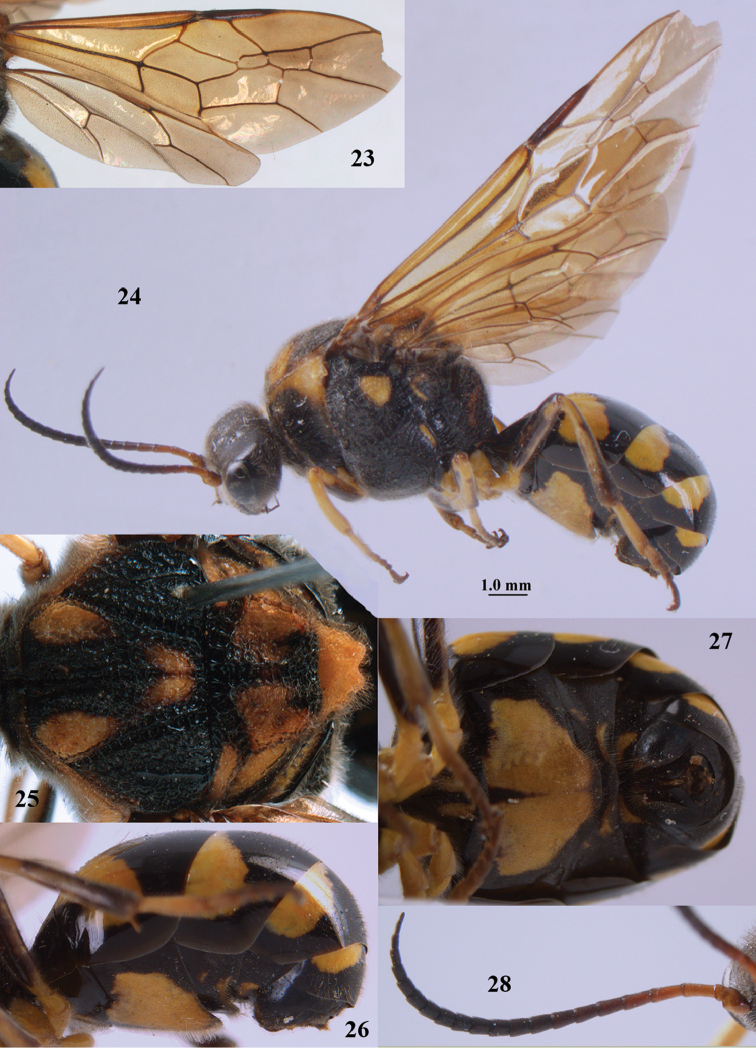
*Bareogonalos jezoensis*, female, Japan (Kyushu, Kobayashi). **23** wings **24** habitus lateral **25** mesosoma dorsal **26** metasoma lateral **27** metasoma ventral **28** antenna lateral.

**Figure 29.**
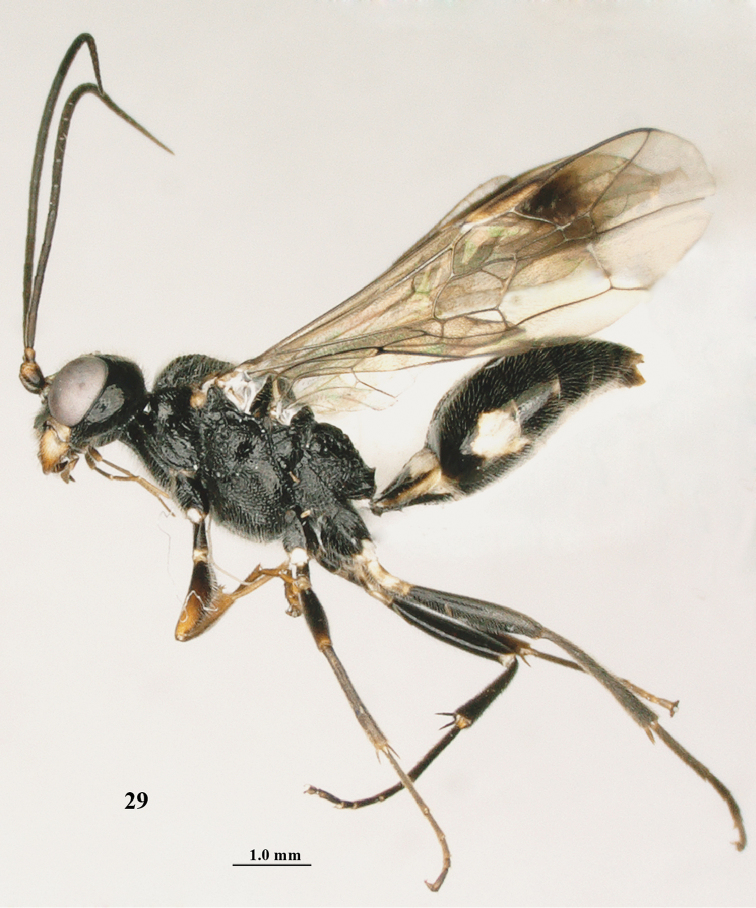
*Jezonogonalos mandibularis* Tan & van Achterberg, sp. n., female, holotype, habitus lateral.

**Figures 30–38.**
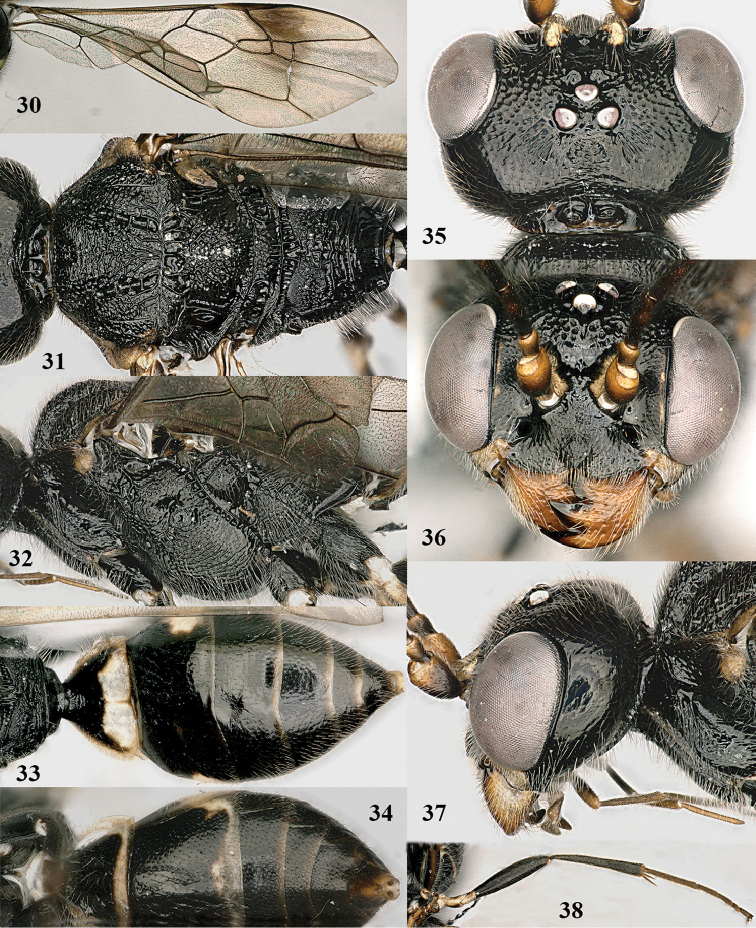
*Jezonogonalos mandibularis* Tan & van Achterberg, sp. n., female, holotype. **30** fore wing **31** mesosoma dorsal **32** mesosoma lateral **33** metasoma dorsal **34** metasoma ventral **35** head dorsal **36** head anterior **37** head lateral **38** hind leg lateral.

### Jezonogonalos shaanxiensis

Taxon classificationAnimaliaHymenopteraTrigonalyidae

Tan & van Achterberg sp. n.

http://zoobank.org/5779AB72-4943-466C-9263-8ECB196F2AF6

Figs 39 , 40–48

#### Type material.

Holotype, ♀ (NWUX) “NW China: Shaanxi, Lower Changqing Re[ser]v[e], Shanshuping Base, 1504 m, 33.67N 107.57E, 23.ix.–10.xi.2016, Y[ellow Malaise] T[rap], Zhao Lin-Peng, NWUX”. Paratypes: 1 ♀ (RMNH), “NW China: Shaanxi, Liping Nat. For. P., MT 1+2, c. 1495 m, 22.vi.–4.ix.2015, 32°47'33"N, 106°39'52"E, JL. Tan & C. v. Achterberg, NWUX”; 1 ♀ (NWUX), “NW. China: Shaanxi, Xunyangba, Ningshan, c. 1300 m, 24.vi. 2011, 33°33'N, 108°32'E, Jiangli Tan, NWUX”.

#### Diagnosis.

**Figure 39.**
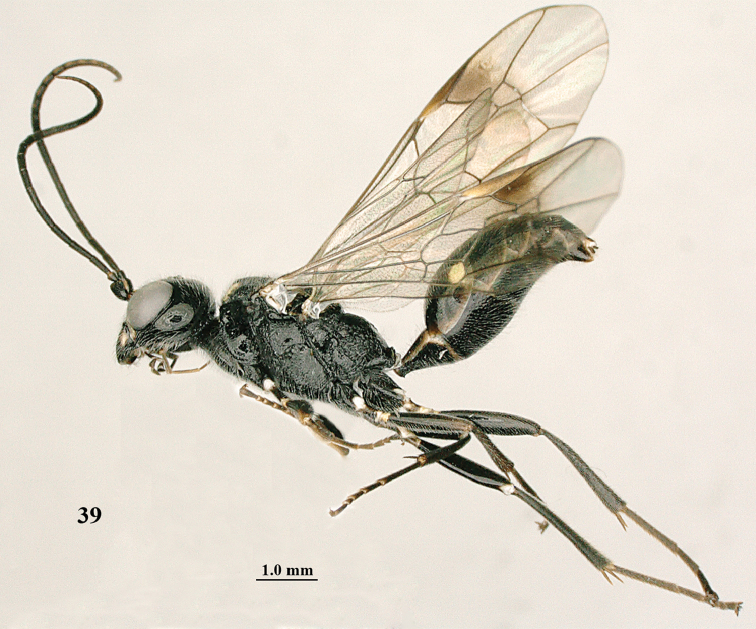
*Jezonogonalos shaanxiensis* Tan & van Achterberg, sp. n., female, holotype, habitus lateral.

Occipital carina very wide medio-dorsally, with pair of curved lamellae separated by carina (Fig. 45); outer side of supra-antennal elevations subvertical, largely smooth, and elevations about 0.8 times as long as scapus (Fig. 45); frons moderately punctate (Fig. 46); supra-antennal elevations largely ivory dorsally (Fig. 45); mandible mainly dark brown, except ivory basal patch and pale brown base of teeth (Fig. 46); metasoma dorsally largely smooth and largely black (Figs 39, 43); first tergite about 0.6 times as long as its apical width (Fig. 43); third sternite about 0.4 times as long as second sternite (Fig. 44). Close to *J. satoi* (Tsuneki) from Taiwan and *J. mandibularis* from Shaanxi, from which the new species differs because of the largely smooth supra-antennal elevations (with carinae or long grooves and some punctures in *J. satoi* and *J. mandibularis*), the densely rugose and rather matt scutellum (largely smooth and shiny), the scutellum somewhat above level of metanotum in lateral view and medially flattened (protruding far above level of metanotum and medially slightly longitudinally depressed), the mandible largely dark brown (largely pale brown and yellowish or ivory); the inner orbita of eye completely ivory (largely dark brown or with incomplete ivory stripe), and the middle lobe of mesoscutum with pair of ivory patches anteriorly (entirely black).

#### Description.

Holotype, ♀, length of body 8.7 mm (of fore wing 7.1 mm).

*Head*. Antenna with 25 segments; frons moderately punctate with smooth interspaces wider than punctures (Fig. 46), with medium-sized whitish setae; vertex smooth, but remotely punctulate (Fig. 45); temple largely smooth, punctulate, but near mandible punctate (Fig. 47); head hardly narrowed behind eyes, eye in dorsal view 1.2 times as long as temple (Fig. 45); occipital carina strongly widened and pair of circular lamellae medio-dorsally, separated by distinct carina (Figs 41, 45), laterally distinctly crenulated; supra-antennal elevations distinctly enlarged (about 0.8 times as long as scapus), largely smooth (except some punctures) and outer side subvertical; clypeus concave and thick medio-ventrally and area above it convex and obtusely protruding (Fig. 46).

*Mesosoma*. Length of mesosoma 1.7 times its height (Fig. 42); mesopleuron antero-dorsally spaced rugose, dorso-posteriorly with some fine striae and remainder smooth and shiny, and antero-ventrally curved rugulose; notauli rather narrow, but deep and coarsely crenulate; middle lobe of mesoscutum transversely rugulose and with some punctures in between, lateral lobes mainly punctate except for a smooth line (Fig. 41); scutellar sulcus wide and coarsely crenulate; scutellum remotely coarsely punctate and laterally with rugae, flattened, in lateral view somewhat above level of metanotum (Fig. 39); metanotum medially evenly convex, shiny and largely smooth (Fig. 41); propodeum antero-laterally irregularly rugose or rugulose and interspaces more or less coriaceous, matt, remainder coarsely transversely rugose and shiny medially, and smooth posteriorly (Fig. 41); posterior propodeal carina thick lamelliform (foramen about 4 times as wide as high medially).

**Figures 40–48.**
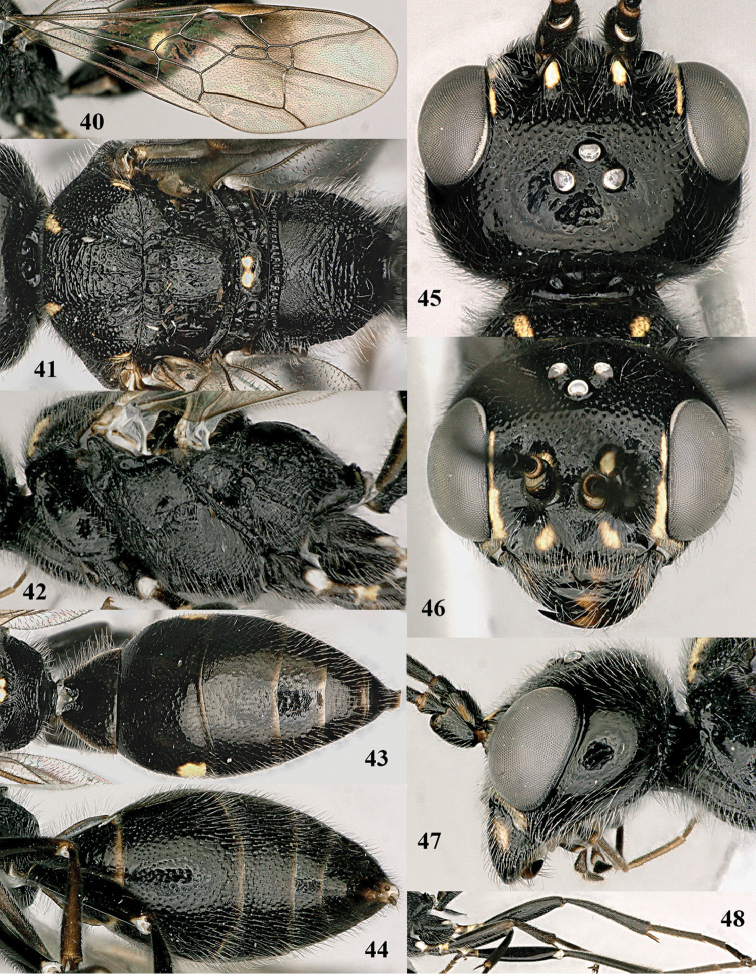
*Jezonogonalos shaanxiensis* Tan & van Achterberg, sp. n., female, holotype. **40** fore wing **41** mesosoma dorsal **42** mesosoma lateral **43** metasoma dorsal **44** metasoma ventral **45** head dorsal **46** head anterior **47** head lateral **48** hind leg lateral.

*Wings.* Fore wing: length of vein 1-M 1.5 times as long as vein 1-SR; third submarginal cell much wider anteriorly than petiolate second cell (Fig. 40).

*Metasoma*. First tergite 0.6 times as long as its apical width, smooth but basal depression anteriorly with some crenulae (Fig. 43); second and following tergites smooth except for punctulation and shiny; sternites rather densely finely punctate, with wide smooth interspaces; second sternite weakly curved in lateral view; third sternite about 0.4 times as long as second sternite (Fig. 44); hypopygium triangularly protruding in ventral view (Fig. 44).

*Colour.* Black; inner orbita narrowly ivory and connected to ivory malar space; pair of patches on clypeus, basal patch of mandible, large patch on supra-antennal elevations, pair of elongate patches on middle lobe of mesoscutum anteriorly, pair of narrow lines near tegulae, pair of small patches on metanotum medially, epipleura of first tergite, large patch apico-laterally on second tergite and narrow apical bands of sternites ivory; 12^th^–22^nd^ antennal segments largely pale brown ventrally; mandible largely dark brown but teeth light brown basally (Figs 46); tegulae and trochanters mainly dark brown; palpi rather dark brown; legs blackish or dark brown, but fore femur apico-ventrally and tibia ventrally yellowish; pterostigma basally yellow, and remainder dark brown; basal half of marginal cell and to a lesser degree area below it dark brown and remainder of wing membrane subhyaline (Fig. 40).

*Variations*. Length of body 8.7–10.2 mm, of fore wing 7.1–8.1 mm; antenna of ♀ with 25(2) or 27(1) segments; metanotum black or with pair of ivory spots medially; ivory patches of clypeus and mesoscutum rather small to large; mesoscutum sometimes with minute ivory spot medio-posteriorly; second submarginal cell of fore wing petiolate or sessile anteriorly; length of vein 1-M of fore wing 1.5–1.7 times as long as vein 1-SR; pterostigma largely yellowish brown or largely dark brown; mandible dark brown or ivory subbasally; whitish setae of mesosoma long or medium-sized.

*Male.* Unknown.

#### Biology.

Unknown.

#### Distribution.

China (Shaanxi). Collected at 1300–1500 m.

#### Etymology.

Named after the province Shaanxi, where it was collected.

### Orthogonalys

Taxon classificationAnimaliaHymenopteraTrigonalyidae

Schulz, 1905

Figs 49 , 50–59 , 60, 61 , 62–70 , 71–79

Orthogonalys Schulz, 1905: 76; Weinstein and Austin 1991: 421; Carmean and Kimsey 1998: 52; Smith and Tripotin 2012: 3; Chen et al. 2014: 60–87 (synonymy, diagnosis, key to Chinese species). Type species (by monotypy): Orthogonalys boliviana Schulz, 1905.

#### Biology.

Reared reared as hyperparasitoid of Tachinidae in caterpillars (Carmean and Kimsey 1998; Murphy et al. 2009). Collected in May–August.

#### Distribution.

Mainly East Palaearctic and Northeast Oriental regions, with few species in East Afrotropical (including Madagascar), Neotropical and Nearctic regions.

#### Key to Chinese species of the genus *Orthogonalys* Schulz

**Table:** 

1	Mesosoma without pale pattern dorsally, at most with an ivory patch on mesoscutum medially (Figs 64, 71); first tergite of ♀ black, largely brownish white or with a reddish yellow patch apically (Figs 66, 74); position of vein 1m-cu of fore wing variable (Figs 60–62)	**2**
–	Mesosoma with linear ivory pattern dorsally (with row of large ivory patches at least on scutellum and metanotum; Fig. 51, rarely with small patches in males); first metasomal tergite of ♀ with brownish or ivory apical transverse band, at most with narrow brown median line; vein 1m-cu of fore wing connected to first submarginal cell (Fig. 50)	**4**
2	Third submarginal cell about 0.3 times as long as second submarginal cell; antenna with a pale brown band subapically (13^th^–15^th^ segments); vein 1m-cu of fore wing connected to second submarginal cell; scutellum sparsely punctate; [only ♂ known; Japan]	***O. fukuiensis* (Tsuneki, 1991)**
–	Third submarginal cell 0.4–0.5 times as long as second submarginal cell (Fig. 51); antenna without a pale band (Figs 60, 61); vein 1m-cu of fore wing connected to first submarginal cell (Fig. 62) or interstitial, rarely connected to second submarginal cell; scutellum moderately to coarsely punctate (Figs 64, 71)	**3**
3	Mesoscutum coriaceous-rugulose and with inconspicuous anterior pair of smooth stripes (Figs 64, 71); area behind stemmaticum with some rugae and posteriorly aciculate (Figs 67, 76); pterostigma of both sexes dark brown (Figs 60–62); frons coarsely punctate (Figs 68, 77)	***O. paraclypeata* sp. n.**
–	Mesoscutum densely to remotely punctate and anterior pair of smooth stripes rather conspicuous; area behind stemmaticum smooth; pterostigma of ♀ largely yellowish brown or brown and of ♂ largely dark brown; frons at most moderately punctate and often largely smooth	***O. clypeata* Chen, van Achterberg, He & Xu, 2014**
4	Temple with large to medium-sized ivory patch (Figs 49, 58), if medium-sized or small then frons laterally widely ivory or yellowish (Fig. 56); third–fifth metasomal tergites of ♀ with pair of well-differentiated large triangular ivory patches; posterior half of third metasomal sternite of ♂ ivory or largely so (Fig. 54); pronotum and mesopleuron often with rich pattern of ivory or pale yellowish patches (Figs 49, 52)	**5**
–	Temple entirely black or with minute pale patch; frons laterally at most with small pale patch; third–fifth tergites of ♀ mainly dark brown, without well-differentiated triangular ivory patches; posterior half of third sternite of ♂ dark brown or largely so; pronotum and mesopleuron entirely black	**7**
5	Vertex with large yellow patch medio-posteriorly; head dorsally and mesoscutum coarsely punctate; posterior half of propodeum obliquely rugose; spiracle of propodeum distinctly protruding; outer side of hind coxa (except base) and hind femur basally and apically yellowish brown; first tergite robust and with median groove	***O. formosana* Teranishi, 1931**
–	Vertex black medio-posteriorly (Fig. 56); head dorsally and mesoscutum largely smooth, punctulate (Figs 51, 56); posterior half of propodeum largely smooth except some transverse rugae; spiracle of propodeum slightly protruding; outer side of hind coxa largely dark brown (Fig. 49); hind femur more or less infuscate or dark brown basally and apically; first tergite less robust and without median groove (Fig. 53)	**6**
6	Temple with vague yellowish patch; yellow or ivory lateral patch of frons widened dorsally; metapleuron with ivory patch; hind femur largely and tibia yellowish brown; supra-antennal elevations largely smooth, punctate	***O. cheni* Chen, van Achterberg, He & Xu, 2014**
–	Temple with well-differentiated yellowish patch (Figs 49, 58); yellow lateral patch of frons parallel-sided or narrowed dorsally (Fig. 56); metapleuron usually entirely black (Figs 49, 52); hind femur (except sometimes medially) and tibia largely dark brown (Figs 49, 59); supra-antennal elevations with coarse rugae (Fig. 56)	***O. hirasana* Teranishi, 1929, re-instated**
7	Length of eye 0.9–1.1 times temple in dorsal view; mesoscutum densely (rugulose-)punctate, hardly shiny and robust in dorsal view; temple often entirely black; basally second sternite of ♀ ivory; posterior half of propodeum with some transverse rugae; fifth and sixth metasomal tergites more or less ivory medio-apically; slightly more robust species (♀); medially third sternite 0.4–0.7 times as long as second sternite (Fig. 73)	***O. robusta* Chen, van Achterberg, He & Xu, 2014**
–	Length of eye 1.2–1.3 times temple in dorsal view; mesoscutum sparsely punctulate, distinctly shiny and rather slender in dorsal view; temple usually with small pale patch; basally second sternite of ♀ dark brown; posterior half of propodeum often largely smooth; slender species (♀♂); medially third sternite 0.6–1.0 times as long as second sternite, rarely less; [first tergite with pair of pale spots (♀) or black (♂); apical half of mandible reddish-brown in Japanese specimens]	***O. elongata* Teranishi, 1929**

#### Notes.

*Orthogonalys centrimaculata* Bischoff, 1951, from N. Vietnam (Sa Pa, Lao Cai) will run in the key to *O. robusta*, but its vertex has a medio-posterior pale patch (absent in *O. robusta*), the mesoscutum is very finely and densely transversely rugulose or coriaceous, matt (mainly finely remotely (rugulose-)punctate and with satin sheen), and hind tibia and tarsus are yellowish brown (dark brown). It runs to *O. formosana* if only the colouration of the vertex is considered, but it has the mesoscutum is very finely and densely transversely rugulose or coriaceous, matt (coarsely punctate and shiny in *O. formosana*) and hind tibia yellowish brown (apical two-thirds dark brown).

### Orthogonalys clypeata

Taxon classificationAnimaliaHymenopteraTrigonalyidae

Chen, van Achterberg, He & Xu, 2014

Orthogonalys clypeata Chen et al., 2014: 67–71 (diagnosis, description, distribution).

#### Material.

3 ♀ + 9 ♂ (NWUX, RMNH), “NW China: Shaanxi, Lower Changqing Re[ser]v[e], Shanshuping, 1445 m, N33°67' E107°57', 18.vi.–17.vii.2016, Y[ellow Malaise] T[rap], Zhao Lin-Peng, NWUX”; 4 ♀ + 6 ♂ (NWUX, RMNH), “NW China: Shaanxi, Xunyangba, Ningshaan, 1481 m, N33°54' E108°55', 1.vii.–17.viii.2016, Y[ellow] and G[reen Malaise] Trap, Jiangli Tan/Qingqing Tan, NWUX”; 9 ♀ + 13 ♂ (NWUX, RMNH), id., but Green Malaise trap, 20.v.–23.vi.2016; 1 ♀ + 1 ♂ (NWUX), “NW China: Shaanxi, Pingheliang, Ningshaan, N33°47' E108°50', B[lack] Mal[aise] trap, 1.vii.–17.viii.2016, 2188 m, J-L. Tan & Q-Q. Tan, NWUX”; 1 ♂ (NWUX), “NW China: Shaanxi, Ningshaan, from Huangguan to Xunyangba, 1236 m, 33°54'N, 105°36'E, 1.vii.–17.viii.2016, black Mal[aise] trap, J-L Tan & Q-Q Tan, NWUX”; 1 ♀ (NWUX), “NW China: Shaanxi, Ningqiang, Hanzhong, Tiankeng, Chanjiyan, N32.46° E106.30°, 25.vi-22.vii.2017, b[lack] Mal. trap, alt. 1638 m, Tan Jiangli, NWUX”.

#### Distribution.

China (Guizhou, Ningxia, Shaanxi, Sichuan, Yunnan). Collected at 1445–1650 m.

#### Notes.

The series shows a considerable variation in the shape of the second and third submarginal cells, but the third cell remains wider anteriorly than the second cell. The size difference is also considerable, e.g., the length of the fore wing (3 ♀) is 4.6–8.2 mm.

### Orthogonalys elongata

Taxon classificationAnimaliaHymenopteraTrigonalyidae

Teranishi, 1929

Orthogonalos elongata Teranishi, 1929: 146; Marshakov 1981: 105; Tsuneki 1991: 20; Weinstein and Austin 1991: 424.
Satogonalos elongata ; Weinstein and Austin 1991: 424.
Orthogonalys elongata ; Carmean and Kimsey 1998: 54; Bennett and Lelej 2003: 8; Chen et al. 2014: 72–80 71 (synonymy, diagnosis, description, distribution).

#### Material.

1 ♀ (NWUX), “NW China: Shaanxi, Ningshaan, from Huangguan to Xunyangba, 1236 m, 33°54'N, 105°36'E, 20.v.–20.vi.2016, black Mal[aise] trap, J-L Tan & Q-Q Tan, NWUX”; 1 ♀ (RMNH), “NW China: Shaanxi, Liping Nat. Forest Park, c. 1490 m, 22.vi.2015, 32°48'N, 106°40'E, Jiangli Tan, NWUX”; 1 ♂ (NWUX), “NW China: Shaanxi, Haopingsi to Dadian, Meixian, Taibai Mt., swept, N34.4, E107.46, 16.vii.2017, alt. 1251 m, Tan Jiangli, NWUX”.

#### Distribution.

China (Henan, *Shaanxi, Sichuan, Tibet); Russia (Far East); Japan (Hokkaido, Honshu).

### Orthogonalys hirasana

Taxon classificationAnimaliaHymenopteraTrigonalyidae

Teranishi, 1929, re-instated

Figs 49 , 50–59

Orthogonalos hirasana Teranishi, 1929: 145; Weinstein and Austin 1991: 424. Synonymized with O. elongata Teranishi, 1929, by Marshakov (1981).
Orthogonalys elongata ; Chen et al. 2014: 72–80 (p.p.).
Orthogonalys albomaculata ? Bischoff, 1951: 908–909.

#### Material.

2 ♀ (NWUX, RMNH), “NW China: Shaanxi, Liping Nat. For. P., MT 1+2, c. 1495 m, 22.vi.–4.ix.2015, 32°47'33"N 106°39'52"E, J-L. Tan & C. v. Achterberg, NWUX”; 2 ♂ (NWUX, RMNH), “NW China: Shaanxi, Lower Changqing Re[ser]v[e], Shanshuping, 1445 m, N33.67 E107.57, 18.vi.–17.vii.2016 & 24.vii–24.viii.2016, Y[ellow Malaise] T[rap], Zhao Lin-Peng, NWUX”; 1 ♂ (NWUX), id., but Shanshuping Base, 1504 m, 25.viii–22.ix.2016; 1 ♀ (NWUX), “NW China: Shaanxi, Ningshaan, from Huangguan to Xunyangba, 1236 m, B[lack] & W[hite Malaise] trap, 17.viii.–5.x.2016, 33°54'N, 105°36'E, J-L. Tan & Q-Q Tan, NWUX”; 1 ♀ (NWUX), “NW China: Shaanxi, Pingheliang, Ningshaan, N33°47' E108°50', B[lack] Mal[aise] trap, 17.viii.–1.x.2016, 2188 m, J-L. Tan & Q-Q. Tan, NWUX”; 1 ♀ + 2 ♂ (NWUX), “NW China: Shaanxi, Ningqiang, Hanzhong, Tiankeng, Chanjiyan, N32.46° E106.30°, 25.vi-22.vii.2017, b[lack] Mal. trap, alt. 1638 m, Tan Jiangli, NWUX”.

#### Distribution.

China (*Shaanxi, *Sichuan), Japan (Honshu), ?India. Collected at 1445–1495 m in China.

#### Notes.

After examination of fresh pale specimens keying out to *Orthogonalys elongata* Teranishi, it was obvious (e.g. colour pattern of head) that they belong to a separate species for which the name of *O. hirasana* Teranishi, 1929, is available. In Chen et al. (2014) the figured female from Sichuan belongs also here considering its colour pattern. Here we illustrate the male of this species (Figs 50–59). The female from Pingheliang has the mesopleuron entirely black.

The interpretation of *O. albomaculata* Bischoff, 1951, from N. India is provisional because only males are known. The propodeum is coarsely reticulate, the pronotal side crenulate medially, the third submarginal cell of fore wing comparatively large and the propodeum has a large ivory patch.

**Figure 49.**
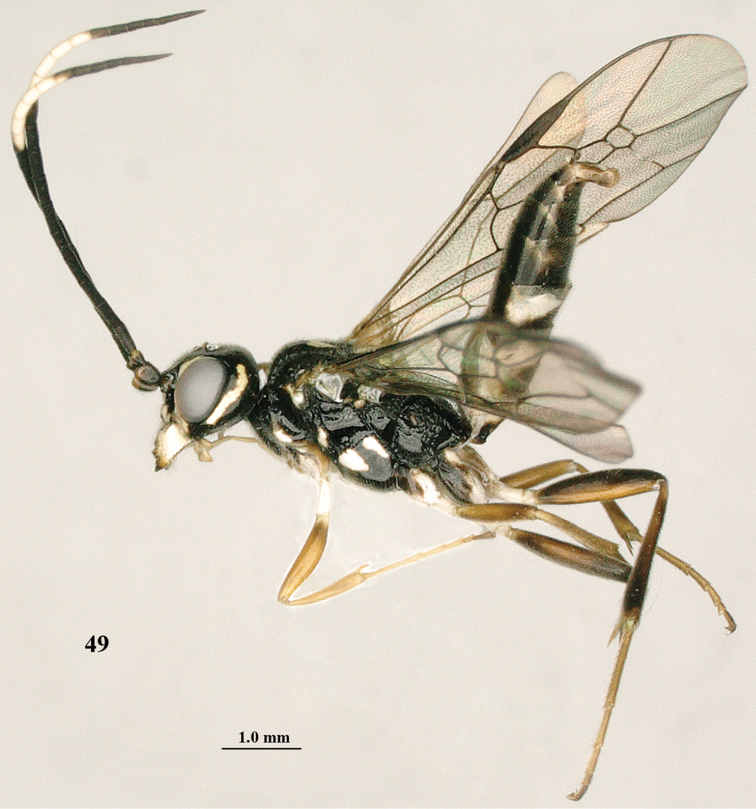
*Orthogonalys hirasana* Teranishi, male, Shaanxi (Shanshuping), habitus lateral.

### Orthogonalys paraclypeata

Taxon classificationAnimaliaHymenopteraTrigonalyidae

Tan & van Achterberg sp. n.

http://zoobank.org/10A80F83-66E2-4AE6-91DD-4534B5B19988

Figs 60–61 , 62–70 , 71–79

#### Type material.

Holotype, ♀ (NWUX), “NW China: Shaanxi, Lower Changqing Re[ser]v[e], Shanshuping, 1445 m, 33.67N 107.57E, 18.vi.–17.vii.2016, Y[ellow Malaise] T[rap], Zhao Lin-Peng, NWUX”. Paratypes: 1 ♂ (NWUX), same data as holotype; 1 ♀ + 8 ♂ (NWUX, RMNH), “NW China: Shaanxi, Xunyangba, Ningshaan, 1481 m, N33°54' E108°55', 1.vii.–17.viii.2016, Y[ellow] and G[reen Malaise] Trap, Jiangli Tan/Qingqing Tan, NWUX”; 2 ♀ + 23 ♂ (NWUX, RMNH), id., but Green Malaise trap (except 1 ♀ from black Malaise trap), 20.v.–23.vi.2016; 3 ♂ (NWUX), “NW China: Shaanxi, Ningshaan, from Huangguan to Xunyangba, 1236 m, 33°54'N, 105°36'E, 1.vii.–17.viii.2016, black Mal[aise] trap, J-L Tan & Q-Q Tan, NWUX”; 4 ♂ (NWUX), id., but 20.v.–20.vi.2016; 2 ♂ (NWUX), “NW China: Shaanxi, Huanghualing, Zhashui, 1408 m, 20.v.–1.vii.2016, 33°80'N, 108°88'E, yellow [Malaise] trap, J-L Tan & Q-Q Tan, NWUX”.

#### Diagnosis.

Antenna without subapical ivory band (Figs 60, 61); occipital carina rather narrow and sparsely crenulate dorsally (Fig. 67); frons moderately shiny and largely coarsely punctate (Fig. 68); area behind stemmaticum with some rugae and posteriorly aciculate (Fig. 67); supra-antennal elevations medium-sized and coarsely punctate (Fig. 67); clypeus strongly convex medially, coarsely punctate and medio-ventrally slightly concave (Fig. 68); basal half of mandible ivory; mesoscutum coriaceous-rugulose and anterior pair of smooth stripes inconspicuous (Fig. 64); scutellum coarsely punctate, longitudinally depressed medially and laterally with some longitudinal rugae, and moderately shiny (Fig. 64); mesosoma without pale pattern or spots dorsally (Fig. 64); third submarginal cell 0.4–0.5 times as long as second submarginal cell, but anteriorly much wider than second cell (Figs 61, 62); pterostigma of both sexes dark brown; anterior 0.8–0.9 of first metasomal tergite and anterior half of second tergite black (Fig. 66).

The new species runs in the key to *Orthogonalys* by Chen et al. (2014) to *O. clypeata* Chen, van Achterberg, He & Xu, 2014, and differs as follows: mesoscutum coriaceous-rugulose and anterior pair of smooth stripes inconspicuous (densely to remotely punctate and anterior pair of smooth stripes rather conspicuous in *O. clypeata*), area behind stemmaticum with some fine rugae and posteriorly aciculate (smooth), frons coarsely punctate (at most moderately punctate and often largely smooth), and pterostigma of ♀ dark brown (largely brownish yellow).

#### Description.

Holotype, ♀, length of body 9.2 mm (of fore wing 7.6 mm).

*Head*. Antenna with 22 segments; frons coarsely punctate; vertex largely smooth laterally, but behind stemmaticum with some fine rugae and posteriorly transversely aciculate (Fig. 67); temple smooth (Fig. 63); head gradually narrowed behind eyes, eye in dorsal view 1.3 times as long as temple (Fig. 67); occipital carina rather narrow and sparsely crenulate dorsally (Fig. 67); supra-antennal elevations medium-sized (about half as long as scapus), outer side subvertical and coarsely densely rugose (Fig. 67); clypeus slightly concave medio-ventrally and strongly convex medially (Fig. 68).

*Mesosoma*. Length of mesosoma 1.5 times its height; mesopleuron below transverse mesopleural groove with some coarse rugae, above groove largely smooth (Fig. 65); transverse mesopleural groove wide, deep and coarsely crenulate; notauli wide, deep and coarsely crenulate; mesoscutum coriaceous-rugulose and anterior pair of smooth stripes of middle lobe inconspicuous, lateral lobe rugulose and with fine punctures (Fig. 64); scutellar sulcus wide and coarsely crenulate; scutellum coarsely punctate, medially shallowly longitudinally depressed and laterally with some longitudinal rugae (Fig. 64); metanotum medially distinctly convex, smooth and shiny but anteriorly rugulose (Fig. 64); propodeum shiny and irregularly spaced rugose (Fig. 64); posterior propodeal carina thick lamelliform and hardly arched, foramen medially 0.3 times higher than wide basally.

*Wings.* Fore wing: length of vein 1-M 2.2 times as long as vein 1-SR (Fig. 62); second submarginal cell twice as long as third cell.

*Metasoma*. First tergite 0.8 times as long as its apical width, smooth and with pair of small depressions medially (Fig. 66); metasoma smooth, but first sternite partly superficially coriaceous (Fig. 69); third sternite about 0.7 times as long as second sternite (Fig. 69); hypopygium triangular (Figs 60, 69).

*Colour.* Black; palpi and tegulae pale yellow; inner orbita (except dorsally) ivory and connected to broadly ivory malar space; basal half of mandible ivory, apical half pale brown, but teeth dark brown; apical quarter of antenna brownish ventrally; first tergite latero-posteriorly, apical half of second tergite (and medio-anteriorly protruding into black area), pair of large triangular spots on third tergite latero-posteriorly, first sternite laterally, second sternite and fourth sternite laterally and medially yellow (Fig. 69); hind trochanter and trochantellus white; coxae and hind femur black; fore and middle trochanters, base of femora, hind tibia and tarsus dark brown; remainder of legs yellowish brown; pterostigma dark brown; wing membrane subhyaline.

*Variations.* Length of body 7.1–9.2 mm, of fore wing 5.8–7.6 mm; antenna with 22(1) or 23(1) segments.

*Male.* Length of body 5.6–10.8 mm, of fore wing 5.9–8.6 mm; antenna with 21(2), 22(15), 23(12), 24(2) segments, apical quarter of antenna brownish ventrally or most of antenna brown; frons densely and coarsely punctate-rugose; clypeus usually entirely black, but sometimes partly or entirely ivory (as in *O. clypeata*); mesoscutum often less distinctly transversely rugose than in females; metasoma darker than of female, dorsally largely black (only apical margin of tergites brownish) but first sternite laterally, second sternite laterally and medio-posteriorly (or brownish yellow with pair of elongate dark patches) and third sternite partly or entirely brownish yellow, but sometimes entirely dark brown; paramere (Figs 61, 75) smaller than of *O. clypeata*.

#### Biology.

Unknown.

#### Distribution.

China (Shaanxi).

#### Etymology.

Named “*paraclypeata*” because it is similar to *O. clypeata* and “para” is Greek for “near”.

### Orthogonalys robusta

Taxon classificationAnimaliaHymenopteraTrigonalyidae

Chen, van Achterberg, He & Xu, 2014

Orthogonalys robusta Chen et al., 2014: 84–87 (diagnosis, description, distribution).

#### Material.

4 ♀ (NWUX, RMNH), “NW China: Shaanxi, Xunyangba, Ningshaan, 1481 m, N33°54' E108°55', 1.vii.–17.viii.2016, Y[ellow] and G [Malaise] Trap, Jiangli Tan, NWUX”; 1 ♂ (NWUX), “NW China: Shaanxi, Huanghualing, Zhashui, 1408 m, 20.v.–1.vii.2016, 33°80'N, 108°88'E, J-L Tan & Q-Q Tan, NWUX”.

#### Distribution.

China (Guangxi, Shaanxi). Collected at 1480–1760 m.

### Pseudogonalos

Taxon classificationAnimaliaHymenopteraTrigonalyidae

Schulz, 1906

Pseudogonalos Schulz, 1906: 209; Weinstein and Austin 1991: 424; Tsuneki 1991: 3; Lelej 1995: 12; Carmean and Kimsey 1998: 72; Chen et al. 2014: 87–95 (synonymy, references, diagnosis, key to Chinese species). Type species (by monotypy): Trigonalis (!) hahnii Spinola, 1840.

#### Biology.

Reared as hyperparasitoid of Ichneumonidae in caterpillars and from Diprionidae (Carmean and Kimsey 1998). Collected in April–August.

#### Distribution.

Palaearctic region, but two species (*P. harmandi* Schulz, 1907 and *P. hahnii* (Spinola)) occur in the North Oriental region.

### Pseudogonalos hahnii

Taxon classificationAnimaliaHymenopteraTrigonalyidae

(Spinola, 1840)

Trigonalis hahnii Spinola, 1840: 1; Weinstein and Austin 1991: 425.
Pseudogonalos hahni (*sic*!); Teranishi 1929: 147; Tsuneki 1991: 14.
Pseudogonalos hahnii ; Marshakov 1981: 104; Lelej 1995: 12; Carmean and Kimsey 1998: 72; Chen et al. 2014: 91–95 (synonymy, references, diagnosis, redescription).

#### Material.

2 ♀ (NWUX, RMNH), “NW. China: Shaanxi, Baolongyu, Mt. Qin, c. 1000 m, 10.vi.2015, 34°03'N, 108°09'E, Jiangli Tan, NWUX”; 1 ♀ (NWUX), “China: Gansu, Xiama, Tianzhu, 16–19.vii.2014, Jiangli Tan”.

#### Distribution.

China (Beijing, Hebei, Henan, Inner Mongolia, Liaoning, *Shaanxi, Yunnan); Russia; Ukraine; Kazakhstan; Mongolia; Western Europe (Lelej 2003). Collected at 0–1000 m.

#### Notes.

Large conspicuous black species with large dark patch near pterostigma of fore wing.

**Figures 50–59.**
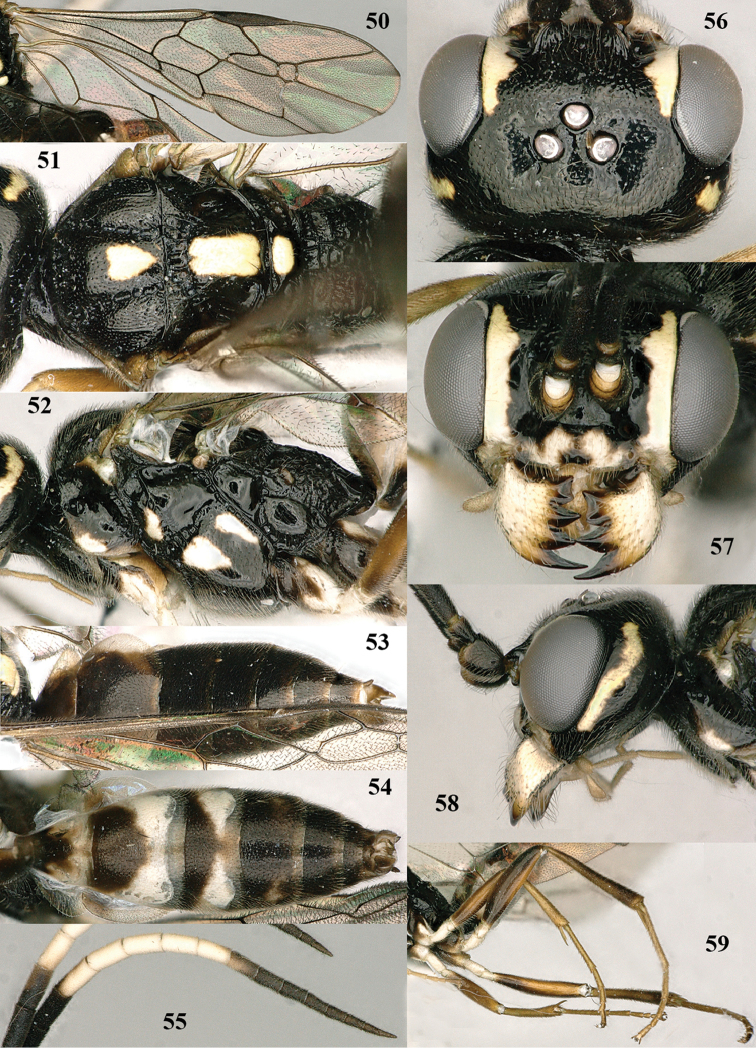
*Orthogonalys hirasana* Teranishi, male, Shaanxi (Shanshuping). **50** fore wing **51** mesosoma dorsal **52** mesosoma lateral **53** metasoma dorsal **54** metasoma ventral **55** apical two-thirds of antenna lateral **56** head dorsal **57** head anterior **58** head lateral **59** hind leg lateral.

**Figures 60, 61.**
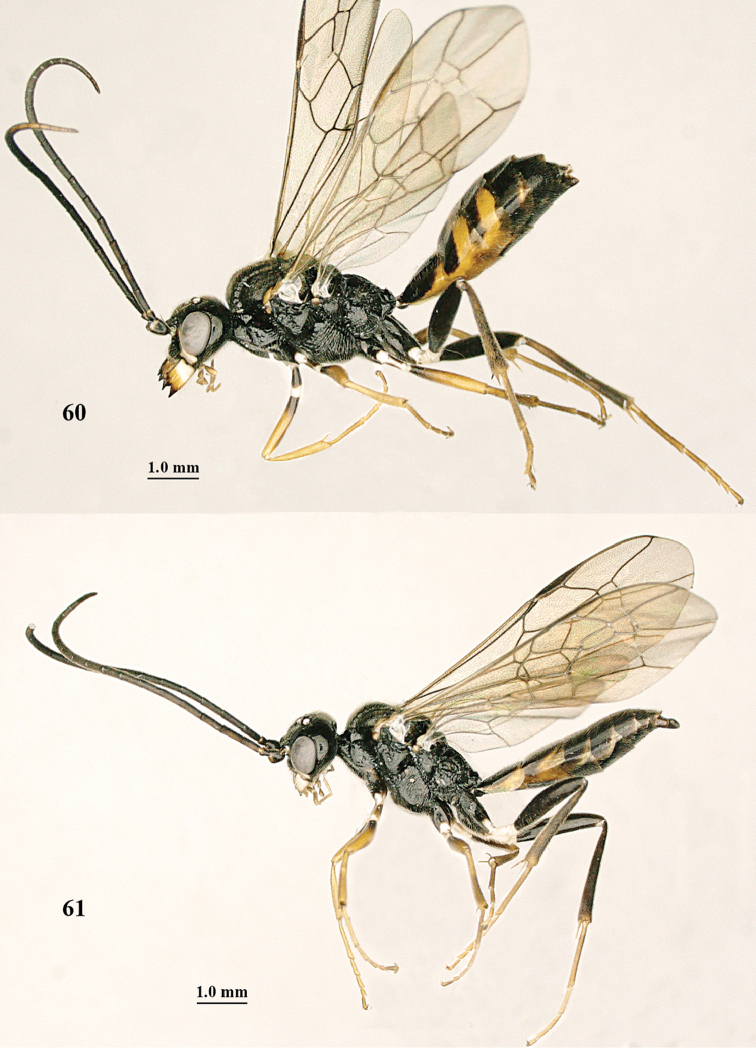
*Orthogonalys paraclypeata* Tan & van Achterberg, sp. n. **60** female, holotype, habitus lateral **61** male, paratype, habitus lateral.

**Figures 62–70.**
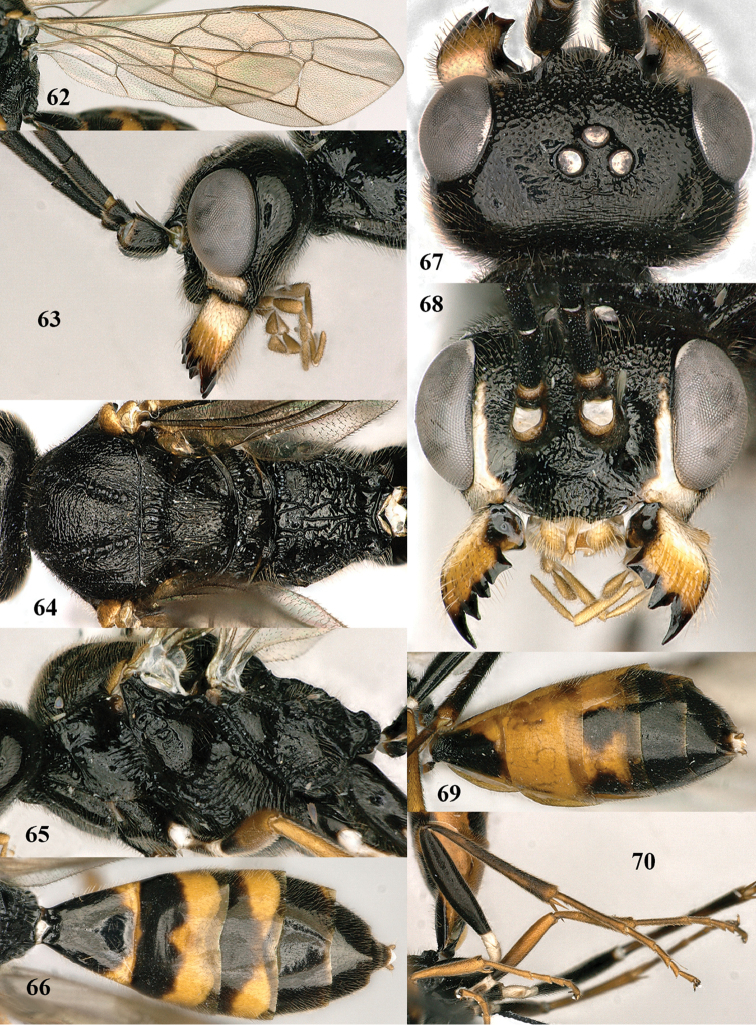
*Orthogonalys paraclypeata* Tan & van Achterberg, sp. n., female, holotype. **62** fore wing **63** head lateral **64** mesosoma dorsal **65** mesosoma lateral **66** metasoma dorsal **67** head dorsal **68** head anterior **69** metasoma ventral **70** hind leg lateral.

**Figures 71–79.**
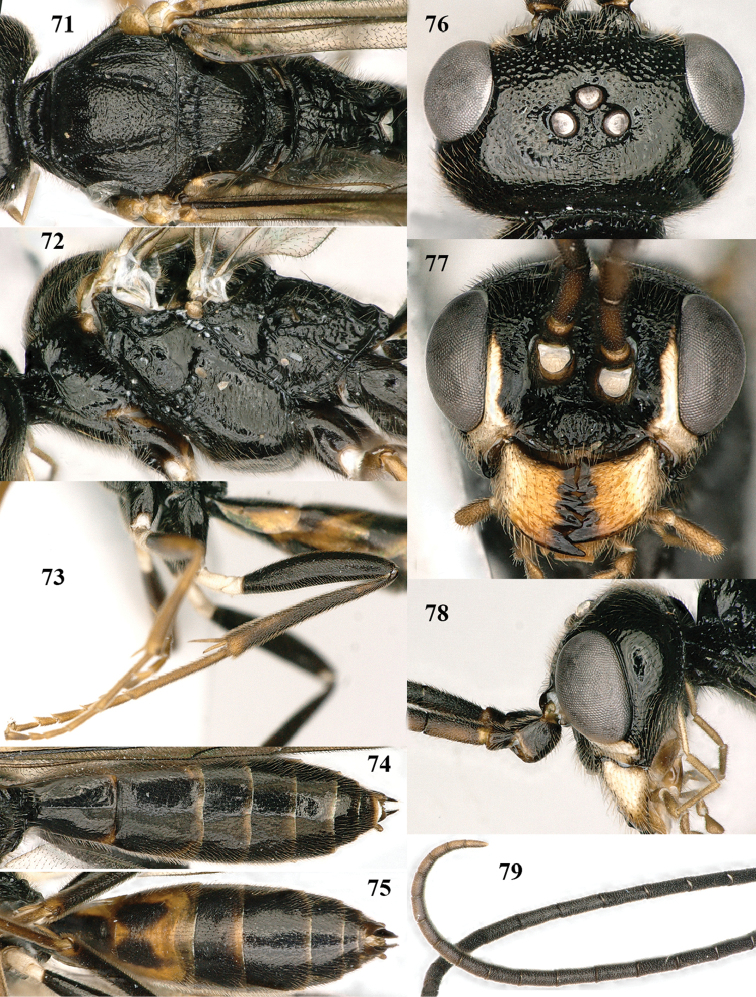
*Orthogonalys paraclypeata* Tan & van Achterberg, sp. n., male, paratype. **71** mesosoma dorsal **72** mesosoma lateral **73** hind leg lateral **74** metasoma dorsal **75** metasoma ventral **76** head dorsal **77** head anterior **78** head lateral **79** apical two-thirds of antenna lateral.

### Taeniogonalos

Taxon classificationAnimaliaHymenopteraTrigonalyidae

Schulz, 1906

Figs 80–81 , 82–91

Taeniogonalos Schulz, 1906: 212; Weinstein and Austin 1991: 416; Tsuneki 1991: 59; Carmean and Kimsey 1998: 65; Chen et al. 2014: 95–193 (synonymy, references, diagnosis, key to Chinese species). Type species (by monotypy): Trigonalys maculata Smith, 1851.

#### Biology.

Reared as hyperparasitoid of parasitoid wasps (Ichneumonidae and Braconidae) and parasitoid flies (Tachinidae) in caterpillars, but some species are primary parasitoids of Pergid sawflies in Australia (Raff 1934; Carne 1969; He and Chen 1986; Weinstein and Austin 1995; Carmean and Kimsey 1998). Collected mainly in April–October, rarely in November or January.

#### Distribution.

This genus occurs in all major regions, but is unknown from Europe and western Nearctic region. Most of the species occur in the East Palaearctic, Northeast Oriental, and Neotropical regions (Carmean and Kimsey 1998).

**Revised part of key to Chinese species of the genus *Taeniogonalos* Schulz**

(for first part, see Chen et al. 2014)

**Table:** 

15	Occipital carina strongly widened medio-dorsally (in ♀ up to 1.5 times as wide as diameter of posterior ocellus) and with pair of circular carinae; third antennal segment of ♀ dark brown ventrally; second sternite of ♀ strongly convex and distinctly punctate medially; anteriorly vertex of ♀ finely punctate and with distinct smooth interspaces; mesosoma of ♀ with limited yellowish pattern or entirely black (as in ♂)	***T. bucarinata* Chen, van Achterberg, He & Xu, 2014**
–	Occipital carina narrow and smooth medio-dorsally, **if** moderately lamelliform (in ♀ up to 0.5 times as wide as diameter of posterior ocellus in some *T. taihorina*) then carina moderately widened medio-dorsally and with one crenula, third antennal segment of ♀ light brown ventrally, and second sternite of ♀ moderately convex and largely smooth medially; sculpture of vertex and colour of mesosoma of ♀ variable	**16**
16	Metasoma dorsally largely orange brown except first tergite and conspicuously densely and long setose; second sternite of ♂ distinctly impressed medio-posteriorly and tricoloured; metanotum of ♂ pale yellow or ivory medially	***T. tricolorisoma* Chen, van Achterberg, He & Xu, 2014**
–	Metasoma dorsally black with yellowish pattern or nearly entirely black, its setosity rather sparse and short to medium-sized; second sternite of ♂ flattened medio-posteriorly and bicoloured or evenly convex and entirely black; metanotum of ♂ black medially or with pair of yellow (often small) patches	**17**
17	Second metasomal sternite of ♀ less convex medially and in lateral view its ventral border gradually sloping posteriorly (Fig. 81), strongly shiny medially **and** third sternite smooth anteriorly or nearly so; second sternite of ♂ evenly slightly convex medio-posteriorly; propodeum comparatively narrow, almost triangular in dorsal view (especially ♂, less so in ♀; Fig. 82) and with nearly straight lateral margins; [vertex largely smooth and often strongly shiny, black, without brownish patch; outer half of lateral mesoscutal lobe usually partly smooth and rather shiny; small species with hind trochanter of ♀ often yellow (Fig. 90), of ♂ more or less darkened]	***T. alticola* (Tsuneki, 1991)**
–	Second sternite of ♀ strongly convex medially and in lateral view its ventral border distinctly sloping posteriorly (Figs 527, 547 in Chen et al. 2014), moderately shiny medially **and** third sternite distinctly punctate anteriorly; second sternite of ♂ slightly flattened medio-posteriorly; propodeum somewhat wider, more oval in dorsal view and lateral margins curved; outer half of lateral mesoscutal lobe distinctly punctate or rugulose, rather matt; vertex distinctly punctate (but less so in small specimens) and moderately shiny	**18**
18	Vertex with elongate brownish patches; third and fourth metasomal tergites very coarsely punctate; metanotum with a pair of yellow spots medially; third sternite coarsely and densely punctate medio-posteriorly (♂) or latero-posteriorly (♀) in front of membranous border; [propodeal foramen comparatively wide and less arched]	***T. formosana*** (Bischoff, 1913)
–	Vertex entirely black or rarely with small brownish patches; third and fourth metasomal tergites usually superficially punctate, rarely nearly as coarse as in *T. formosana*; metanotum black medially and at most with a pair of lateral yellow spots; third sternite usually more spaced and finer punctate posteriorly	***T. taihorina* (Bischoff, 1914)**

### Taeniogonalos alticola

Taxon classificationAnimaliaHymenopteraTrigonalyidae

(Tsuneki, 1991)

Figs 80–81 , 82–91

Taiwanogonalos alticola Tsuneki, 1991: 42. Synonymized with Taeniogonalos maga (Teranishi, 1929) by Carmean and Kimsey 1998 and re-instated as valid species by Chen et al. (2014).
Taeniogonalos alticola ; Chen et al. 2014: 101–108 (description, diagnosis, distribution).
Taiwanogonalos alishana Tsuneki, 1991: 36. Synonymized by Carmean and Kimsey 1998 with T. maga. **Syn. n.**

#### Material.

4 ♀ (NWUX), “NW China: Shaanxi, Lower Changqing Re[ser]v[e], Shanshuping, 1445 m, N33.67 E107.57, 18.vi.–17.vii.2016, Y[ellow Malaise] T[rap], Zhao Lin-Peng, NWUX”; 4 ♀ + 8 ♂ (NWUX, RMNH), “NW. China: Shaanxi, Xunyangba, Ningshaan, 1481 m, Y[ellow] and G[reen Malaise] trap, 1.vii.–17.viiii.2016, 33°33'N, 108°32'E, Jiangli Tan/Qingqing Tan, NWUX”; 3 ♂ (NWUX), id., but Green Malaise trap, 20.v.–23.vi.2016; 1 ♀ (NWUX), “NW China: Ningxia, Liupan Mt., Jingyuan, Dongshanpo For[est] Farm, N35°23'26” E106°20'34.27”, 4.viii.2015, c 1800 m, Jiangli Tan, NWUX”; 1 ♀ (NWUX), “NW. China: Shaanxi, Panda valley, Foping, 1411 m, black Mal[aise] trap, 1.vii–18.viii.2016, 33°67'N, 107°97'E, Jiangli Tan, NWUX”; 1 ♂ (NWUX), “NW China: Shaanxi, Huanghualing, Zhashui, 1408 m, 20.v.–1.vii.2016, 33°80'N, 108°88'E, yellow [Malaise] trap, J-L Tan & Q-Q Tan, NWUX”; 1 ♀ (NWUX), “NW China: Shaanxi, Ningshaan, from Huangguan to Xunyangba, 1236 m, 33°54'N, 105°36'E, 1.vii.–17.viii.2016, black Mal[aise] trap, J-L Tan & Q-Q Tan, NWUX”; 1 ♀ (NWUX), “NW China: Shaanxi, Liping Nat. For. P., MT 1+2, c. 1495 m, 22.vi.–4.ix.2015, 32°47'33"N 106°39'52"E, JL. Tan & C. v. Achterberg, NWUX”; 1 ♀ + 2 ♂ (NWUX): “NW China: Shaanxi, Ningqiang, Hanzhong, Tiankeng, Chanjiyan, N32.46° E106.30°, 25.vi-22.vii.2017, b[lack] Mal. trap, alt. 1638 m, Tan Jiangli, NWUX”. First report of female and new for continental China.

#### Description.

Illustrated ♀ from Liupan Mt., length of body 5.6 mm (of fore wing 5.1 mm).

*Head*. Antenna with 20 segments; frons coarsely punctate, with smooth interspaces narrower than punctures and with some rugae anteriorly (Fig. 88); vertex largely smooth and strongly shiny posteriorly, but spaced moderately punctate anteriorly (Fig. 87); temple smooth except some punctures near eye and mandible (Fig. 89); head gradually narrowed behind eyes, eye in dorsal view 1.1 times as long as temple (Fig. 87); occipital carina narrow, non-lamelliform and smooth dorsally (Fig. 87); supra-antennal elevations medium-sized (about half as long as scapus), outer side subvertical anteriorly and with few rather small punctures and apically with few striae (Fig. 87); clypeus slightly concave medio-ventrally and with blunt tubercle above it.

*Mesosoma*. Length of mesosoma 1.5 times its height; propleuron largely rugose; mesopleuron below transverse mesopleural groove rugulose, above groove densely rugose but posteriorly largely smooth (Fig. 84); transverse mesopleural groove wide, deep and coarsely crenulated; mesosternum mainly transversely aciculate; notauli rather wide, deep and coarsely crenulated; middle lobe of mesoscutum transversely rugulose, but posteriorly with some rugae and anterior pair of smooth stripes rather conspicuous, lateral lobe with coarse rugae but laterally largely smooth or superficially rugulose (Fig. 83); scutellar sulcus rather wide and coarsely crenulated; scutellum reticulate-rugulose and medially slightly longitudinally depressed laterally (Fig. 83); metanotum rugose and medially flattened, but submedially convex (Fig. 83); propodeum slender, lateral sides nearly straight, its surface shiny, finely rugose anteriorly and superficially rugulose posteriorly (Fig. 83); posterior propodeal carina narrow lamelliform medially and strongly arched, foramen medially 0.4 times higher than wide basally.

*Wings.* Fore wing: length of vein 1-M 1.7 times as long as vein 1-SR (Fig. 82); second submarginal cell 1.3 times as long as third cell.

*Metasoma*. First tergite 0.6 times as long as its apical width, smooth and medially largely depressed (Fig. 85); metasoma smooth and strongly shiny, third–fifth tergites superficially punctulate (Fig. 86); second sternite strongly shiny and convex; second–fourth sternites superficially spaced punctulate or finely punctate; third sternite slightly concave and about 0.3 times as long as second sternite (Fig. 86).

*Colour.* Black; palpi dark brown basally and pale brown apically; antenna pale brown, but scapus and apical 0.4 of antenna darkened; inner orbita slightly brownish near level of antennal sockets; mandible mainly dark brown, but medially pale brown; sixth tergite yellowish ivory; robust hind trochanter and trochantellus mainly white except some slightly brownish small patches; fore and middle tibiae and tarsi brownish yellow, but middle tibia dark brown posteriorly; remainder of legs, tegulae and pterostigma dark brown; wing membrane subhyaline except for infuscated patch near apex of fore wing (Fig. 82).

#### Notes.

The holotype of *T. alishana* fits better with *T. alticola* than with *T. taihorina* considering its sculpture. The male holotype has been collected at the same day and locality as the male holotype of *T. alticola*.

#### Distribution.

China (*Ningxia, *Shaanxi, Taiwan). Collected at 1236–1800 m.

### Taeniogonalos bucarinata

Taxon classificationAnimaliaHymenopteraTrigonalyidae

Chen, van Achterberg, He & Xu, 2014

Taeniogonalos bucarinata Chen, van Achterberg, He & Xu, 2014: 108–113 (description, diagnosis, distribution).

#### Material.

2 ♂ (NWUX, RMNH), “NW. China: Shaanxi, Xunyangba, Ningshan, c. 1300 m, 2.vi. & 30.ix.2014, 33°33'N, 108°32'E, Jiangli Tan, NWUX”; 4 ♀ + 16 ♂ (NWUX, RMNH), id., but 1481 m, yellow or black Malaise trap, 1.vii.–17.viiii.2016; 9 ♂ (NWUX), id., but 20.v.–23.vi.2016, green Malaise trap, J-L. Tan & Q-Q. Tan; 1 ♀ (NWUX), id., but black trap; 1 ♀ (NWUX), id., but yellow/green Malaise trap, 17.viii.–3.x.2016; 2 ♂ (NWUX), “NW. China: Shaanxi, Qinling Mts, Foping, near Biol. Stat., c. 1400 m, Mal. trap, 29.v.–19.vi.2016, 33°40'N, 107°58'E, J-L. Tan & C. v. Achterberg, NWUX”; 1 ♂ (NWUX), “NW China: Shaanxi, Liping Nat. For. P., MT 1+2, c. 1495 m, 22.vi–4.ix.2015, 32°47'33"N 106°39'52"E, JL. Tan & C. v. Achterberg, NWUX”; 2 ♀ (NWUX, RMNH), “NW China: Shaanxi, Pingheliang, Ningshaan, N33°47' E108°50', B[lack] Mal[aise] trap, 17.viii.–1.x.2016, 2188 m, J-L. Tan & Q-Q. Tan, NWUX”; 4 ♂ (NWUX), “NW China: Shaanxi, Huanghualing, Zhashui, 1408 m, 20.v.–1.vii.2016, 33°80'N, 108°88'E, yellow [Malaise] trap, J-L Tan & Q-Q Tan, NWUX”; 1 ♀ + 1 ♂ (RMNH), “NW China: Shaanxi, Liangfengya, Foping, 1729 m, 19.viii.–13.xi.2016, 33°69'N, 107°90'E, y[ellow] Mal[aise] trap, J-L Tan & Q-Q Tan, NWUX”; 4 ♂ (NWUX), “NW China: Shaanxi, Ningshaan, from Huangguan to Xunyangba, 1236 m, 33°54'N, 105°36'E, 1.vii.–17.viii.2016, black Mal[aise] trap, J-L Tan & Q-Q Tan, NWUX”; 1 ♀ (NWUX), id., but 20.v.–20.vi.2016; 1 ♀ (NWUX), “NW China: Shaanxi, Upper Changqing Re[ser]v[e], Shanshuping, 1556 m, N33.67 E107.58, 24.vii–24.viii.2016, Y[ellow Malaise] T[rap], Zhao Lin-Peng, NWUX”; 2 ♂ (NWUX), “NW. China: Shaanxi, Qinling Mts, Luoyuan, c. 1350 m, 16.iv.–28.v.2016, 34°12'N, 109°50'E, J-L Tan & C. v. Achterberg”; 1 ♀ (NWUX), “NW China: Ningxia, Liupan Mt., Jingyuan, Erlonghe For[est] Farm, N35°23'24.14" E106°20'41.43", 2.viii.2015, c 1800 m, Jiangli Tan, NWUX”; 3 ♀ + 6 ♂ (NWUX): “NW China: Shaanxi, Ningqiang, Hanzhong, Tiankeng, Chanjiyan, N32.46° E106.30°, 25.vi-22.vii.2017, b[lack] Mal. trap, alt. 1638 m, Tan Jiangli, NWUX”.

#### Distribution.

China (Fujian, Gansu, Henan, Ningxia, Shaanxi, Sichuan, Yunnan, Zhejiang). Collected at 1200–2350 m.

### Taeniogonalos fasciata

Taxon classificationAnimaliaHymenopteraTrigonalyidae

(Strand, 1913)

Poecilogonalos fasciata Strand, 1913: 97; Weinstein and Austin 1991: 422; Lelej 1995: 14; He and Lou 2001: 686; He 2004: 73.
Taeniogonalos fasciata ; Carmean and Kimsey 1998: 67; Chen et al. 2014: 117–125 (synonymy, description, diagnosis, distribution).

#### Material.

1 ♂ (NWUX), “NW China: Shaanxi, Ningshaan, from Huangguan to Xunyangba, 1236 m, 33°54'N, 105°36'E, 20.v.–20.vi.2016, black Mal[aise] trap, J-L Tan & Q-Q Tan, NWUX”

#### Distribution.

China (Anhui, Fujian, Guangdong, Guangxi, Guizhou, Hainan, Henan, Hunan, Jilin, Liaoning, Shaanxi, Taiwan, Zhejiang); Russia (Primorskii krai); Japan; Korea. Collected at 990–1550 m. Also reported from Iran, Malaysia, and Indonesia but these reports need confirmation.

### Taeniogonalos formosana

Taxon classificationAnimaliaHymenopteraTrigonalyidae

(Bischoff, 1913)

Poecilogonalos formosana Bischoff, 1913: 151; Tsuneki 1991: 51; Weinstein and Austin 1991: 423.
Taeniogonalos formosana ; Carmean and Kimsey 1998: 67; Chen et al. 2014: 129–138 (description, diagnosis, distribution).

#### Material.

1 ♀ + 4 ♂ (NWUX, RMNH), “NW. China: Shaanxi, Xunyangba, Ningshan, 1481 m, Y[ellow] and G[reen Malaise] trap, 1.vii–17.viiii.2016, 33°33'N, 108°32'E, Jiangli Tan/Qingqing Tan, NWUX”; 3 ♂ (NWUX, RMNH), id., but Green Malaise trap, 20.v.–23.vi.2016; 2 ♂ (NWUX), “NW. China: Shaanxi, Panda valley, Foping, 1411 m, black Mal[aise] trap, 1.vii–18.viii.2016, 33°67'N, 107°97'E, Qingqing Tan, NWUX”; 74 ♂ (NWUX, RMNH), “NW China: Shaanxi, Ningqiang, Hanzhong, Tiankeng, Chanjiyan, N32.46° E106.30°, 25.vi-22.vii.2017, b[lack] Mal. trap, alt. 1638 m, Tan Jiangli, NWUX”.

#### Notes.

The specimens from Shaanxi belong to the form *intermedia* (Chen, 1949) because the hind trochanters (as often fore trochanters) are entirely yellow and with tri-coloured (yellow, black and brown) second tergite and sternite latero-posteriorly. Colour of both mesosoma and head are very variable, e.g. the scutellum is black in most specimens but sometimes it has two yellow patches.

#### Distribution.

China (Fujian, Guangdong, Guizhou, Henan, Jilin, Ningxia, *Shaanxi, Shanxi, Sichuan, Taiwan, Tibet, Yunnan, Zhejiang); Russia (Far East); Japan. Collected at 800–1638 m.

### Taeniogonalos taihorina

Taxon classificationAnimaliaHymenopteraTrigonalyidae

(Bischoff, 1914)

Nanogonalos taihorina Bischoff, 1914: 93; Tsuneki 1991: 58; Weinstein and Austin 1991: 421.
Taeniogonalos taihorina ; Carmean and Kimsey 1998: 68; Chen et al. 2014: 171–179 (synonymy, diagnosis, distribution).
Poecilogonalos maga Teranishi, 1929: 148; Marshakov 1981: 106; Tsuneki 1991: 51; Weinstein and Austin 1991: 423; Lelej 1995: 14. **Syn. n.**
Taeniogonalos maga ; Chen et al. 2014: 146–150 (synonymy, diagnosis, distribution).
Taiwanogonalos claripennis Tsuneki, 1991: 38. Synonymized by Carmean and Kimsey 1998 with T. maga. **Syn. n.**

#### Material.

5 ♀ (NWUX, RMNH), “NW China: Shaanxi, Lower Changqing Re[ser]v[e], Shanshuping, 1445 m, N33.67 E107.57, 18.vi.–17.vii.2016, Y[ellow Malaise] T[rap], Zhao Lin-Peng, NWUX”; 1 ♀ (NWUX), id., but Shanshuping Base, 1504 m, 23.ix.–10.xi.2016; 1 ♀ (NWUX), “NW China: Shaanxi, Liping Nat. For. P., MT 1+2, c. 1495 m, 22.vi.–4.ix.2015, 32°47'33"N 106°39'52"E, JL. Tan & C. v. Achterberg, NWUX”; 6 ♀ (NWUX, RMNH), “NW. China: Shaanxi, Xunyangba, Ningshan, 1481 m, Y[ellow] and G[reen Malaise] trap, 1.vii.–17.viiii.2016, 33°33'N, 108°32'E, Jiangli Tan/Qingqing Tan, NWUX”; 2 ♀ + 4 ♂ (NWUX), id., but 17.viii.–3.x.2016; 2 ♀ + 2 ♂ (NWUX), id., but 20.v.–23.vi.2016, Green trap; 1 ♂ (NWUX), id., but Black Malaise trap; 3 ♀ (NWUX), “NW China: Shaanxi, Ningshaan, from Huangguan to Xunyangba, 1236 m, 33°54'N, 105°36'E, 1.vii.–17.viii.2016, black Mal[aise] trap, J-L Tan & Q-Q Tan, NWUX”; 1 ♀ (NWUX), “NW. China: Shaanxi, Panda valley, Foping, 1411 m, black Mal[aise] trap, 1.vii.–18.viii.2016, 33°67'N, 107°97'E, Jiangli Tan, NWUX”; 1 ♀ + 1 ♂ (NWUX): “NW China: Shaanxi, Ningqiang, Hanzhong, Tiankeng, Chanjiyan, N32.46° E106.30°, 25.vi-22.vii.2017, b[lack] Mal. trap, alt. 1638 m, Tan Jiangli, NWUX”.

#### Notes.

In the key to Chinese *Taeniogonalos* spp. (Chen et al. 2014) *T. maga* (Teranishi) is retained as valid species because of the more lamelliform occipital carina. The occipital carina of the holotype of *T. taihorina* (Bischoff) is narrow medio-dorsally (similar to latero-dorsally) and not lamelliform. After examining the series from Shaanxi it is obvious that the width of the occipital carina varies from dorsally as wide as laterally and hardly or not lamelliform to about twice as wide as laterally and distinctly lamelliform. We failed to find additional characters to separate both taxa and, therefore, we synonymize both species.

#### Distribution.

China (Fujian, Gansu, Guangxi, Heilongjiang, Hubei, Ningxia, *Shaanxi, Sichuan, Taiwan, Tibet, Yunnan, Zhejiang); Russia (Far East); Japan (Hokkaido, Honshu). Collected at 500–2530 m.

### Taeniogonalos tricolor

Taxon classificationAnimaliaHymenopteraTrigonalyidae

(Chen, 1949)

Poecilogonalos tricolor Chen, 1949: 16; Weinstein and Austin 1991: 424; He and Lou 2001: 687.
Paecilogonalos (!) tricolor; He 2004: 75.
Taeniogonalos tricolour (!): Carmean and Kimsey 1998: 68.
Taeniogonalos tricolor ; Chen et al. 2014: 182–186 (description, diagnosis, distribution).

#### Material.

2 ♂ (NWUX, RMNH), “NW. China: Shaanxi, Panda valley, Foping, 1411 m, black Mal[aise] trap, 1.vii–18.viii.2016, 33°67'N, 107°97'E, Jiangli Tan, NWUX”; 1 ♂ (NWUX): “NW China: Shaanxi, Ningqiang, Hanzhong, Tiankeng, Chanjiyan, N32.46° E106.30°, 25.vi-22.vii.2017, b[lack] Mal. trap, alt. 1638 m, Tan Jiangli, NWUX”.

#### Distribution.

China (Henan, Fujian, Guangxi, Guizhou, Hainan, Hubei, Jiangxi, Shaanxi, Sichuan, Yunnan, Zhejiang); Korea; Laos; Thailand. Collected at 900–2000 m.

### Teranishia

Taxon classificationAnimaliaHymenopteraTrigonalyidae

Tsuneki, 1991

Teranishia Tsuneki, 1991: 15–18; Lelej 1995: 12, 2003: 3; Carmean and Kimsey 1998: 73; Chen et al. 2014: 193–201 (diagnosis, key to species). Type species (by monotypy): Teranishia nipponica Tsuneki, 1991.

#### Biology.

Unknown. Collected in June–September.

#### Distribution.

China, Japan.

### Teranishia crenulata

Taxon classificationAnimaliaHymenopteraTrigonalyidae

Chen, van Achterberg, He & Xu, 2014

Teranishia crenulata Chen, van Achterberg, He & Xu, 2014: 194–197 (diagnosis, description).

#### Material.

3 ♂ (NWUX, RMNH), “NW China: Ningxia, Liupan Mt., Jingyuan, Erlonghe For[est] Farm, N35°23'24.14” E106°20'41.43”, Mal[aise] tr[ap], 2–5.viii.2015, c 1800 m, Jiangli Tan, NWUX”.

#### Distribution.

China (Gansu, Ningxia, Sichuan). Collected at 1800–2539 m.

### Teranishia glabrata

Taxon classificationAnimaliaHymenopteraTrigonalyidae

Chen, van Achterberg, He & Xu, 2014

Teranishia glabrata Chen, van Achterberg, He & Xu, 2014: 197–201 (diagnosis, description).

#### Material.

2 ♀ (NWUX, RMNH), “NW China: Shaanxi, Pingheliang, Ningshaan, N33°47' E108°50', B[lack] Mal[aise] trap, 17.viii.–1.x.2016, 2188 m, J-L. Tan & Q-Q. Tan, NWUX”.

#### Distribution.

China (Gansu, Ningxia, *Shaanxi, Sichuan). Collected at 1400–2188 m.

**Figures 80, 81.**
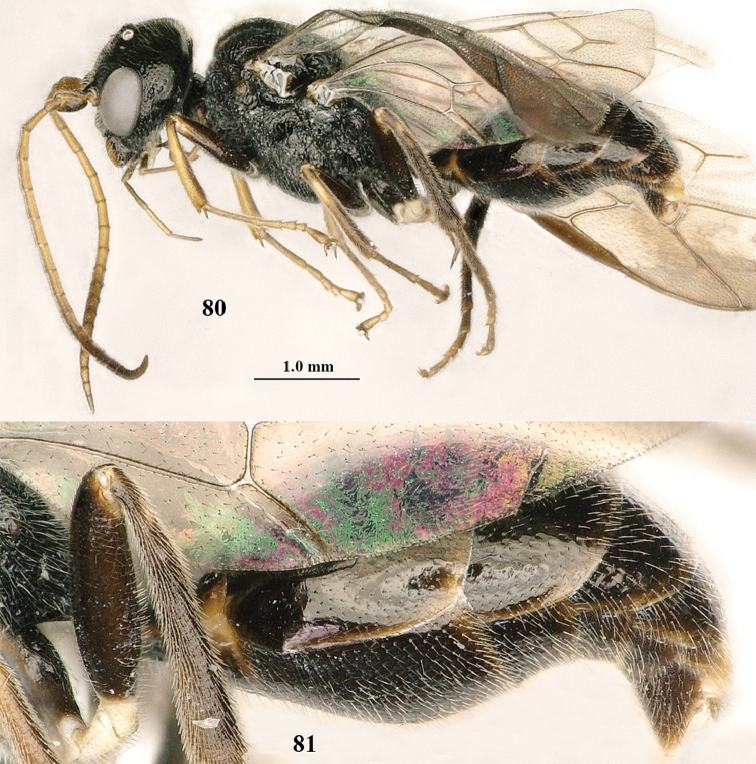
*Taeniogonalos alticola* (Tsuneki), female, Ningxia (Liupan Mt.). **80** habitus lateral **81** metasomal lateral.

**Figures 82–91.**
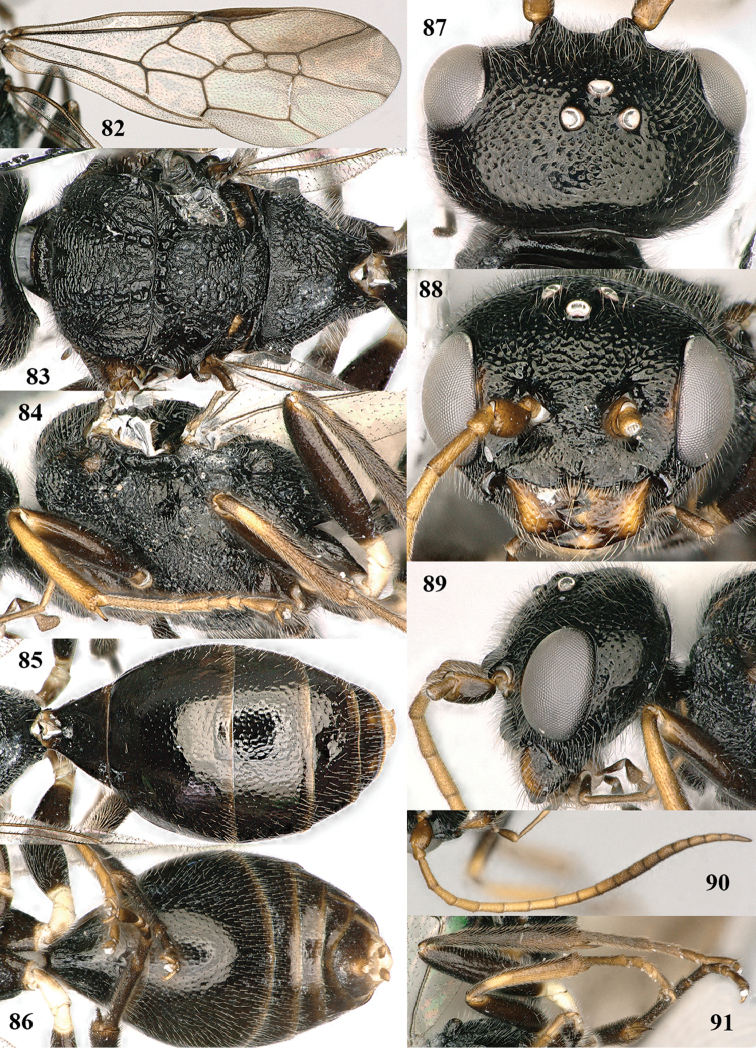
*Taeniogonalos alticola* (Tsuneki), female, Ningxia (Liupan Mt.). **82** fore wing **83** mesosoma dorsal **84** mesosoma lateral **85** metasoma dorsal **86** metasoma ventral **87** head dorsal **88** head anterior **89** head lateral **90** antenna lateral **91** hind leg lateral.

## Supplementary Material

XML Treatment for Trigonalyidae

XML Treatment for Bareogonalos

XML Treatment for Bareogonalos xibeidai

XML Treatment for Jezonogonalos

XML Treatment for Jezonogonalos luteata

XML Treatment for Jezonogonalos mandibularis

XML Treatment for Jezonogonalos shaanxiensis

XML Treatment for Orthogonalys

XML Treatment for Orthogonalys clypeata

XML Treatment for Orthogonalys elongata

XML Treatment for Orthogonalys hirasana

XML Treatment for Orthogonalys paraclypeata

XML Treatment for Orthogonalys robusta

XML Treatment for Pseudogonalos

XML Treatment for Pseudogonalos hahnii

XML Treatment for Taeniogonalos

XML Treatment for Taeniogonalos alticola

XML Treatment for Taeniogonalos bucarinata

XML Treatment for Taeniogonalos fasciata

XML Treatment for Taeniogonalos formosana

XML Treatment for Taeniogonalos taihorina

XML Treatment for Taeniogonalos tricolor

XML Treatment for Teranishia

XML Treatment for Teranishia crenulata

XML Treatment for Teranishia glabrata
